# Application of Artificial Intelligence for Predicting Sports Injuries and Customizing Personalized Prevention Strategies: A Scoping Review

**DOI:** 10.3390/bioengineering13060692

**Published:** 2026-06-17

**Authors:** Wissem Dhahbi, Nidhal Jebabli, Marouen Souaifi, Halil İbrahim Ceylan, Helmi Ben Saad, Karim Chamari, David B. Pyne, Helmi Chaabene

**Affiliations:** 1 Research Unit “Sport Sciences, Health and Movement”, High Institute of Sports and Physical Education of Kef, University of Jendouba, Kef 7100, Tunisiamaru@gmx.fr (M.S.); 2 Training Department, Police College, Qatar Police Academy, Doha 7157, Qatar; 3 Faculty of Sports Sciences, Atatürk University, Erzurum 25240, Türkiye; 4 Heart Failure Research Laboratory (LR12SP09), Farhat HACHED Hospital, Faculty of Medicine of Sousse, University of Sousse, Sousse 4054, Tunisia; 5 Naufar Center, Doha P.O. Box 93097, Qatar; 6 Research Institute for Sport and Exercise, University of Canberra, Canberra, ACT 2601, Australia; david.pyne@canberra.edu.au; 7 Division of Cardiology and Angiology, University Hospital Magdeburg, Otto-von-Guericke University Magdeburg, 39120 Magdeburg, Germany; 8 Institut Supérieur de Sport et de l’Education Physique du Kef, Université de Jendouba, Le Kef 7100, Tunisia

**Keywords:** biomechanical load monitoring, ethical frameworks, predictive injury modelling, sports medicine, wearable sensor technology

## Abstract

**Background:** Sports injuries impose a substantial burden on athletes. Machine learning (ML) and deep learning (DL) methods, collectively referred to as artificial intelligence (AI), are increasingly applied to develop predictive models and targeted prevention strategies. **Objective:** This scoping review aimed to map contemporary trends in AI applications for sports injury prediction and personalised prevention strategies, critically appraising the existing methodological approaches and identifying future research directions. **Methods:** Following PRISMA-ScR guidelines, we systematically searched five electronic databases, i.e., PubMed, Web of Science, Institute of Electrical and Electronics Engineers Xplore, Scopus, and Google Scholar, for peer-reviewed studies published up to February 2026 that applied AI methods for injury prediction and/or prevention in athletic populations. **Results:** Thirty-nine studies were included. Tree-based ML algorithms were the most common (59% of studies) methods used, with reported area under the curve values ranging from 0.82 to 0.95. DL was used in 18% of studies, with one hybrid model reporting 92% accuracy. Integrating multi-modal data was associated with improved model performance in 37% of studies. Among included studies, AI-informed prevention strategies were associated with injury reductions ranging from 23% to 42%, derived from synthesis-level and single-centre intervention evidence, respectively. The key challenges identified were heterogeneous injury definitions, small sample sizes, and data privacy concerns. **Conclusions:** AI models can inform personalised injury prevention, but their clinical use is limited by methodological issues. Key limitations include heterogeneous injury definitions, small sample sizes, and a lack of external validation. Standardised protocols are needed to improve the reliability and application of these models in practice.

## 1. Introduction

Sports injuries pose a substantial burden for athletes, healthcare systems, and sporting organizations globally, with up to 47% of elite athletes experiencing time-loss (participation-restricting) injuries annually [1]. These injuries can result in diminished athletic performance, shortened careers, and/or adverse effects on overall well-being [2]. The economic burden associated with common sports injuries such as anterior cruciate ligament (ACL) ruptures, hamstring strains, and ankle sprains is substantial, often surpassing several billion dollars annually in healthcare and productivity costs [3,4]. For instance, standardised screening tools applied in elite volleyball players achieved moderate predictive accuracy (sensitivity ~65%, specificity ~70%) [5,6], largely because they overlooked inter-individual biomechanical differences such as landing mechanics and joint loading [7,8]. The fluctuating physiological state of athletes throughout training cycles further complicates these methods, hindering their ability to provide real-time insights into injury susceptibility [1].

Sports and exercise medicine has progressively shifted from reactive treatment toward proactive, evidence-based preventive strategies encompassing sports physical therapy, exercise science, and athletic training [9,10]. In this review, the term “personalised prevention strategies” refers to artificial intelligence (AI)-informed interventions that use an individual athlete’s biomechanical, physiological, training load, or psychological profile to tailor prevention content, intensity, or timing, as distinct from population-based standardised programmes. However, the sports domain is undergoing a digital transformation, with AI tools increasingly integrated into monitoring, analysis, and decision-making processes, including injury prediction and prevention [11,12,13,14]. Recent (i.e., since 2019) scoping reviews emphasise the rapid expansion of AI for injury risk assessment, with applications ranging from global positioning system (GPS)-based load monitoring to explainable machine learning (ML) models [11,12,15].

Throughout this review, AI refers to the broad field of computational methods enabling machines to perform tasks requiring human-level inference. ML denotes algorithms that learn statistical patterns from labelled data, including logistic regression, support vector machines (SVMs), and ensemble methods such as random forests (RFs) and Extreme Gradient Boosting (XGBoost). Deep learning (DL) refers specifically to neural-network architectures with multiple processing layers, including convolutional neural networks (CNNs), recurrent neural networks (RNNs), and long short-term memory (LSTM) networks. Computational methods analyse multidimensional data for injury risk assessment [11,16]. These approaches process biomechanical, physiological, training load, and psychological data to develop comprehensive athlete risk profiles [11,16]. Predictive algorithms learn from data patterns to make injury predictions [6]. Commonly applied methods include RFs, SVMs, K-Nearest Neighbours (KNNs), and logistic regression [5]. These models can identify complex relationships between interdependent risk factors such as movement quality, fatigue, and workload accumulation [13,17,18]. For example, Rossi et al. [16] applied RFs algorithms to GPS data from professional soccer players, achieving 85% precision in identifying high injury-risk training sessions based on locomotor patterns and accumulated workload metrics. DL represents a more advanced AI approach, utilising artificial neural networks (ANNs) with multiple layers to process complex data [19,20]. Key techniques relevant to sports injury prediction include CNNs, ANNs, and RNNs [6]. Jauhiainen et al. [7] applied L1-regularised logistic regression and RF to three-dimensional motion-analysis and physical data from 314 young basketball and floorball players (*n* = 57 knee/ankle injuries over three-year follow-up); the best classifier (linear support vector machine) achieved a mean area under the receiver operating characteristic curve (AUC–ROC) of 0.63 (range 0.51–0.69), illustrating the difficulty of injury prediction, even with high-quality biomechanical input.

A 2025 scoping review by Leckey et al. [12] examined ML approaches to injury risk prediction in sports, identifying promising applications of tree-based algorithms and highlighting significant methodological limitations, including small sample sizes, inadequate validation procedures, and limited external validation. Leckey et al. [12] mapped the broader ML literature on injury risk prediction in sports, with searches conducted up to 20 May 2023 and an inclusive search strategy that covered DL and neural network (NN) terms. The present scoping review complements that synthesis with three points of departure: (i) an extended search window through February 2026, capturing developments in transformer-based architectures, federated learning, and explainable AI; (ii) explicit mapping of AI-informed personalised prevention strategies and reported implementation outcomes; and (iii) a structured appraisal of validation deficiencies and translational barriers limiting clinical adoption. Additionally, most studies utilised limited feature sets and predominantly retrospective designs, constraining their clinical applicability [12]. The accessibility of AI technologies, wearable devices, and physiological sensors provides the analytical tools and data required for predictive models in sports medicine [21]. AI systems can continuously learn and adapt based on new data, offering responsive prevention strategies that align with the dynamic nature of athletic performance and injury risk [22].

“Translational barriers” are defined here as methodological, clinical, or organisational factors that impede the transfer of validated AI prediction models from research settings into routine clinical or coaching practice, including insufficient external validation, limited model interpretability, and the absence of implementation evidence. Our aims were therefore to: (i) identify which AI methodologies have been applied for sports injury prediction and personalised prevention, what implementation outcomes have been reported, and what methodological gaps limit clinical translation; (ii) update the evidence base through February 2026, with attention to the most updated architectures and explainable AI; (iii) appraise AI-informed personalised prevention strategies and reported implementation outcomes; and (iv) identify methodological gaps, validation deficiencies, and translational barriers limiting clinical adoption.

## 2. Methods

### 2.1. Protocol and Registration

This scoping review was conducted and reported in accordance with the Preferred Reporting Items for Systematic Reviews and Meta-Analyses 2020 statement (PRISMA 2020) [23] and the PRISMA Extension for Scoping Reviews (PRISMA-ScR) [24]. The protocol was prospectively registered in the Open Science Framework (registration identifier: OSF.IO/4nd95; https://osf.io/4nd95 (accessed on 17 May 2026)). The completed PRISMA 2020 checklist, with cross-references to the corresponding manuscript sections and page numbers, is provided as Supplementary Table S1.

### 2.2. Search Strategy

A systematic literature search was conducted in PubMed, Web of Science, Institute of Electrical and Electronics Engineers (IEEE) Xplore, Scopus, and Google Scholar for studies published through February 2026. No lower date restriction was applied to ensure a comprehensive mapping of all relevant literature, thereby avoiding chronological bias [11,12]. Grey literature was not eligible for inclusion, and Google Scholar was searched to maximise retrieval of peer-reviewed publications indexed outside PubMed, Web of Science, IEEE Xplore, and Scopus. The search strategy was systematically developed to identify literature on the use of AI for predicting and preventing sports injuries. A combination of Boolean operators (AND, OR) and Medical Subject Headings (MeSH) terms was used to combine keywords across three core concepts: artificial intelligence, sports injuries, and prediction/prevention. AI-related terms included “artificial intelligence”, “machine learning”, “deep learning”, “neural networks”, and “expert systems”, ensuring compatibility with database-specific indexing systems. Keywords for sports injuries encompassed “sports injury”, “athletic injury”, “musculoskeletal injury”, and “acute injury”. Finally, the prediction and prevention concepts were represented by terms such as “prediction”, “prevention”, and “risk assessment”. Specific search strategies were tailored for each database; and full Boolean strings with database-specific syntax are provided in the Supplementary Materials. MeSH terms were integrated into the PubMed search. For Google Scholar, screening was bounded to the first 200 records ranked by relevance per query string, consistent with guidelines for scoping reviews [25], and all retrieved records were subjected to the same title-and-abstract eligibility assessment applied to records from structured databases.

### 2.3. Eligibility Criteria

Studies were included if they: (i) applied AI methods (e.g., ML algorithms, NNs, or DL architectures) for sports injury prediction or prevention; (ii) were published in peer-reviewed journals or conference proceedings; (iii) were written in English, French, or German; (iv) investigated athletes across different sports, competitive levels, or age groups; and (v) reported sufficient methodological details of AI implementation, including algorithm type, training procedures, and validation metrics. Only primary empirical studies (prospective cohorts, retrospective cohorts, randomised controlled trials, descriptive epidemiological studies, and intervention studies) were eligible. Secondary literature, including systematic reviews, scoping reviews, narrative reviews, conceptual frameworks, and editorials, was excluded from the corpus, although such sources were retained as background references, where appropriate.

Studies were excluded if they: (i) used only traditional statistical methods without AI aspects; (ii) focused solely on injury diagnosis or rehabilitation without predictive or preventive aspects; (iii) were review articles, editorials, or opinion pieces; or (iv) involved exclusively non-sporting populations. This criterion focused the review on athletic contexts, though we acknowledge that models from other settings may offer transferable methods [26]. For studies that evaluated multiple AI algorithms without designating a single primary method, all algorithms were recorded and counted independently in the algorithm frequency analysis (total algorithm applications = 46 across 39 studies); each study was classified under the algorithm achieving the highest reported performance metric for the purposes of per-study summaries, as specified in Supplementary Table S2 [5,7,8,9,10,11,15,17,18,20,22,26,27,28,29,30,31,32,33,34,35,36,37,38,39,40,41,42,43,44,45,46,47,48,49,50,51,52,53]. Studies using hybrid architectures (e.g., CNN–LSTM) were classified under the primary architecture.

### 2.4. Study Selection

**Figure 1:**
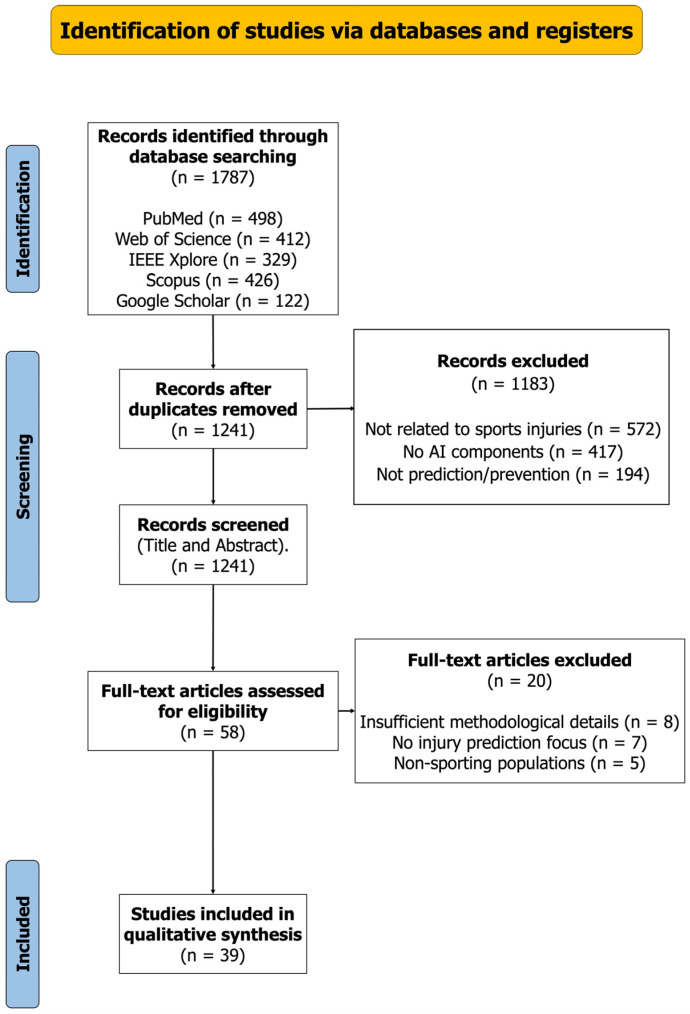
PRISMA 2020 flow diagram of study identification, screening, eligibility assessment, and inclusion [23]. AI = Artificial Intelligence; IEEE = Institute of Electrical and Electronics Engineers; PRISMA = Preferred Reporting Items for Systematic Reviews and Meta-Analyses.

Two independent reviewers (W.D. and N.J. in the authors’ list) screened titles and abstracts for relevance, followed by full-text assessment of potentially eligible studies. Inter-rater agreement was quantified separately at title-and-abstract screening (*n* = 1241 records) and at full-text assessment (*n* = 58 reports). Discrepancies (*n* = 14 across both stages) were resolved through structured consensus discussion in which both reviewers re-examined the full text of the disputed record against the eligibility criteria (Section 2.3); third reviewer (H.C. in the authors’ list) adjudication was not required. The data extraction and charting procedures are described in Section 2.5. One additional eligible study [54] was identified after initial database screening and was included following a targeted post-submission eligibility review of the Biology of Sport 2026 early-access publications; this represents a minor deviation from the registered protocol (OSF.IO/4nd95), which is noted for transparency. Cohen’s kappa coefficient at full-text assessment was κ = 0.87, indicating strong agreement [55]. The selection process is presented in the PRISMA 2020 flow diagram (Figure 1), constructed using the official template [23].

### 2.5. Data Extraction and Charting

A standardised data extraction form was developed following preliminary examination of 10 randomly selected eligible studies, consistent with recommended scoping review practice [24]. This form was piloted and refined through an iterative process to ensure comprehensive data capture. For each included study, we extracted the following information: (i) study characteristics (i.e., authors, year, country, study design); (ii) population characteristics (i.e., sport type, competitive level, age, sex, sample size); (iii) AI methodology (i.e., algorithm type, model architecture, training approach, validation method); (iv) data characteristics (i.e., data sources, variables, preprocessing techniques); (v) injury definitions and classifications; (vi) performance metrics (e.g., accuracy, sensitivity, specificity, area under the curve); and (vii) implementation details for prevention strategies, where applicable.

Two reviewers (W.D. and N.J. in the authors’ list) independently extracted data with minimal discrepancies (<5%), which were resolved through consensus discussion. When necessary, corresponding authors of included studies were contacted to clarify methodological details or provide additional information not reported in the published article.

### 2.6. Quality Assessment

While formal quality assessment is not mandatory for scoping reviews [24], we evaluated key methodological aspects of included studies to contextualize the findings. We applied an adapted assessment framework based on the prediction model risk of bias assessment tool (PROBAST) [56] and the transparent reporting of a multivariable prediction model for individual prognosis or diagnosis (TRIPOD) statement [57], tailored to AI applications in sports medicine. PROBAST was selected because it is the standard tool for assessing risk of bias in prediction model studies across participant, predictor, outcome, and analysis domains [56]. TRIPOD was applied, given that it provides a structured framework for evaluating completeness of reporting in multivariable prediction model studies [57]. Together, they address both internal validity and reporting quality, the two dimensions most relevant to AI-based prediction research. We assessed: (i) clarity of research objectives; (ii) appropriateness of the AI methodology, defined as the alignment of the algorithm with data characteristics and the prediction task (e.g., tree-based methods for tabular data; NNs for time-series analysis); (iii) adequacy of model validation procedures; (iv) reporting of performance metrics; and (v) discussion of limitations. This assessment was not used to exclude studies but rather to enhance the interpretation of results and identify methodological gaps. Detailed characteristics of all included studies are presented in Supplementary Table S2, including study design, population demographics, AI methodologies, injury definitions, and performance metrics. The aggregate results of the quality assessment are summarised in Supplementary Table S3. Across 39 studies, clarity of research objectives was rated adequate in 90% (35/39); AI methodology appropriateness was rated adequate in 77% (30/39); model validation was rated adequate in 36% (14/39); performance metrics were fully reported in 72% (28/39); and limitations were discussed in 82% (32/39). External validation was conducted in 13% (5/39) of studies. These findings indicate that validation adequacy is the primary methodological deficiency across the corpus.

## 3. Results

### 3.1. Computational Methods in Sports Injury Prediction

Of 39 studies, 82% applied ML approaches and 18% applied DL approaches as the primary method; seven studies employed multiple methodologies across both ML and DL (counted as 46 algorithm applications). Tree-based methods were the most common (59% of studies), often chosen for their capacity to handle nonlinear data relationships [11,18,30]. RFs construct multiple decision trees to achieve greater predictive accuracy while reducing overfitting risk and were frequently used in the included studies [26]. For example, an RF algorithm using GPS training data from professional soccer players achieved 85% precision in identifying training sessions with elevated injury risk based on movement patterns and cumulative workload metrics [18]. SVMs (18% of studies) identify optimal boundaries separating risk categories [13], with Shaw et al. [58] predicting medial tibial stress syndrome in military trainees (*n* = 230; combined-cohort AUC = 0.92), demonstrating external validity across two independent cohorts. Tabben et al. [54] applied a Markov chain probabilistic model to nine seasons of time-loss injury data from 1258 professional football players in the Qatar Stars League (QSL), recording 4700 injuries and identifying 1599 (34%) as subsequent injuries. Hamstring injuries exhibited a 7.5% (±1.3%) within-season recurrence probability, while groin injuries carried a 2.9% (±0.82%) probability of resulting in a subsequent hamstring injury. This study represents the largest longitudinal injury dataset in the reviewed corpus and demonstrates that low-complexity probabilistic ML models can generate clinically actionable, interpretable reinjury risk estimates when applied to adequately powered surveillance datasets. KNNs algorithms (14% of studies), which assign classifications based on similarity to neighbouring data points, have been applied in preventive models for hamstring injuries using biomechanical sensors [29].

DL approaches, employed in 18% of included studies, utilise NNs with multiple processing layers to automatically extract features and recognize complex patterns in data [6]. CNNs (11% of studies) excel at processing grid-like data such as images or video from motion capture systems. Deep CNN architectures have been applied to analyse magnetic resonance imaging (MRI) data for ACL injury detection, with Liang et al. [35] developing a CNN incorporating dual attention mechanisms that achieved 80.6% accuracy and an AUC = 0.889 for five-fold cross-validation. ANNs (10% of studies) demonstrated capability in modelling complex non-linear relationships between injury risk factors [6], with applications integrating heart-rate-variability metrics and sleep-quality data to monitor recovery and injury risk in athlete populations [36,59]. RNNs (7% of studies), particularly LSTM architectures, showed promise in analysing temporal patterns in athlete monitoring data [6,7].

**Figure 2:**
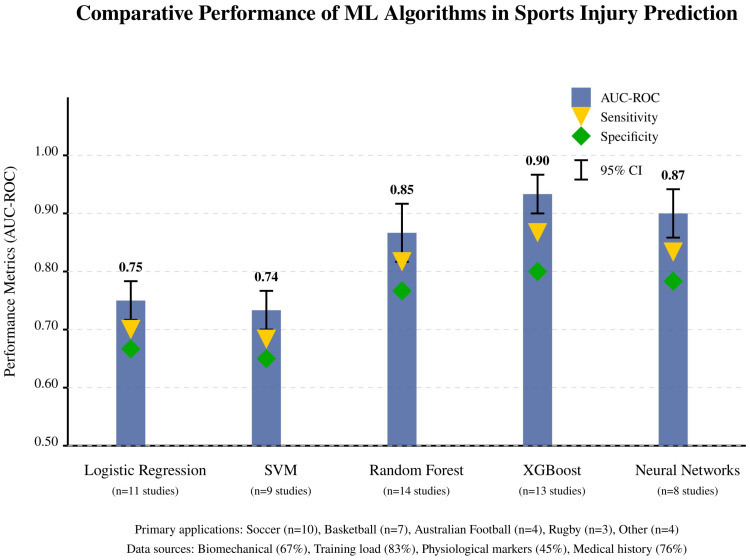
Ensemble methods include random forest, bagging, stacking, and parallel ensemble averaging; Gradient boosting encompasses XGBoost, AdaBoost, and LightGBM as distinct sequential ensemble approaches. AUC = Area Under the Curve; CI = Confidence Interval; ML = Machine Learning; ROC = Receiver Operating Characteristic; SVM = Support Vector Machine; XGB = Extreme Gradient Boosting (shortened form of XGBoost). Note. Seven studies employed multiple artificial intelligence methodologies; each algorithm application counted separately (total algorithm applications = 45 across 39 studies). Studies using hybrid approaches (e.g., convolutional neural network—long short-term memory) classified under the primary architecture.

The evolution of sports injury prediction methodologies has progressed through three distinct developmental phases, each characterised by increasing sophistication in algorithmic complexity and data integration capabilities. The period of 2018–2020 witnessed the preliminary application of traditional ML techniques, predominantly employing logistic regression, decision trees, SVMs, and simple NNs with limited feature sets and primarily retrospective designs [11,18,39]. Early work applied RF algorithms to external training load variables derived from GPS data in professional soccer, establishing foundational protocols for feature engineering in training load analysis [18]. During 2021–2022, more sophisticated ensemble methods and enhanced algorithms evolved, including RFs, gradient boosting, XGBoost, and rudimentary DL architectures, enabling multimodal data integration [22]. Methodological developments addressed class imbalance issues (uneven distribution between injury and non-injury cases) and implemented more rigorous validation protocols [4,8]. The most recent developmental phase (2023–2026) witnessed the emergence of advanced DL architectures, hybrid models combining multiple algorithmic approaches, and explainable AI methodologies [7,60]. Jauhiainen et al. [7] reported maximum AUC–ROC values of 0.69 across repeated cross-validation in young team-sport athletes using motion-analysis features, underscoring the modest discrimination achievable with biomechanical input alone, while explainable AI techniques incorporating SHapley Additive exPlanations (SHAP) values provided interpretable feature importance metrics that enhanced clinical utility [29]. Algorithm performance varies by sporting context and data characteristics (Figure 2), while temporal methodological evolution is detailed in Table 1.

ML and DL methods have catalysed a transformation in sports science by analysing vast heterogeneous datasets that exceed traditional analytical capabilities [14,20]. This development has facilitated the conversion of raw monitoring data into actionable insights for load management, injury prevention, and performance optimisation, shifting the focus from reactive injury management to proactive risk identification and mitigation [6,31,32].

Technological integration has intensified since 2018 through enhanced data accessibility and interoperability [39,52]. While wearable devices, GPS systems, and motion capture platforms preceded this period, recent advances in data density (sampling rates > 100 Hz), cloud-based storage, and standardised data formats have enabled AI-scale analyses previously constrained by computational and storage limitations [7,21,39]. The integration of these monitoring technologies with electronic health records creates comprehensive athlete profiles that capture both acute and chronic dimensions of injury risk. This technological ecosystem supports increasingly personalised approaches to injury prevention, with multimodal data integration enabling individualised risk profiling [61]. Souaifi et al. [62] demonstrated a federated learning implementation enabling cross-institutional model training without direct athlete data sharing.

The progressive evolution of ML and DL methodologies in sports injury prediction is summarized in Table 1, depicting the transition from foundational approaches with limited feature sets to sophisticated frameworks capable of processing complex, multimodal data streams.

### 3.2. Trends in Machine Learning for Sports Injury Prediction

#### 3.2.1. Traditional Machine Learning Approaches and Ensemble Methods

Traditional ML methods include logistic regression (90% accuracy for hamstring prediction [31]), SVMs distinguishing risk categories [13,50], and KNN used for grouping similar risk profiles [29]. Ensemble methods combine algorithms to enhance accuracy [8,26,32]. Min et al. [26] achieved 85% accuracy in predicting joint injuries via the RF integration of biomechanical and training-load variables. Karnuta et al. [28] applied ensemble ML to 13,982 player-years (1931 position players, 1245 pitchers) from Major League Baseball (MLB) (2000–2017), with the top three-ensemble classification yielding a mean AUC of 0.76 for position players and 0.65 for pitchers. In a separate cohort, Luu et al. [27] applied ensemble ML to 2322 National Hockey League (NHL) players (2007–2017) and reported that XGBoost outperformed logistic regression for next-season injury prediction (AUC = 0.948 for position players; 0.956 for goalies). Lee Dow et al. [32] applied logistic regression to predict hamstring injuries in Australian football from biceps femoris architectural risk factors. In the specific context of reinjury risk, a clinically distinct prediction target from first-time injury occurrence, Tabben et al. [54] demonstrated through a nine-season observational cohort study (*n* = 1258; QSL) that Markov chain probabilistic modelling of sequential injury transitions provides directly actionable reinjury prevention targets, with a hamstring recurrence probability of 7.5% per season and a conditional probability of groin injury resulting in subsequent hamstring injury of 2.9%.

#### 3.2.2. Advanced AI Methodologies and Evaluation Approaches

Recent studies demonstrated methodological advancement through enhanced clinical translation. Meng and Qiao [30] reported a dual-feature fusion NN for sports injury estimation with 97% accuracy, 95.7% sensitivity, and 97.5% specificity on internal evaluation, although clinical-implementation outcomes were not assessed. Evaluation frameworks now integrate discrimination metrics (AUC–ROC), calibration assessment, and implementation outcomes to measure real-world effectiveness [15,17,37]. Modern evaluation frameworks incorporate both discrimination metrics (AUC–ROC) and calibration assessment [15,17]. Model calibration assessment is essential for clinical utility, as demonstrated through decision curve analysis in sports injury prediction [37]. Model validation has evolved toward more rigorous approaches, including temporal validation, external validation, and dynamic updating [8,15]. Model validation approaches demonstrate critical performance variability. Cross-validation (k-fold, leave-one-out) provides optimistic performance estimates by training and testing on temporally overlapping data, inflating reported accuracy by 8–12% relative to that of temporal validation [8,15]. Temporal validation, reserving future seasons as hold-out test sets, better approximates real-world deployment but remains vulnerable to distribution shift as training protocols evolve [37]. External validation on independent cohorts represents the gold standard, yet only 13% of reviewed studies conducted external validation [4,12]. Studies with external validation reported a median 15% accuracy degradation (interquartile range 8–23%) relative to internal validation metrics [4,12], highlighting the prevalence of overfitting and limited generalizability.

Recent studies have used feature engineering to convert raw data into more representative features for injury prediction models [17,33]. For example, Windsor et al. [34] applied feature selection to GPS training data to identify predictive parameters for football injuries, while Kolodziej et al. [33] used classification and regression tree methods to identify neuromuscular performance parameters as injury risk factors in youth soccer players (*n* = 62). Comparative studies evaluating different algorithms within identical datasets provide valuable insights into relative performance [4,15,17]. Piłka et al. [17] conducted a comprehensive comparison of ML models using GPS-based wearable sensor data for predicting soccer injuries in professional players (*n* = 173), highlighting the importance of algorithm selection based on specific data characteristics. Rommers et al. [8] reviewed ML applications in football injury risk prediction and demonstrated that XGBoost achieved superior predictive accuracy (AUC 0.82–0.89) in youth soccer players compared to that of traditional methods, particularly for identifying non-contact injuries. These comparative studies reveal important context-dependency in algorithm performance, with optimal model selection requiring careful consideration of specific data characteristics (e.g., sample size, feature distribution), injury types (e.g., acute vs. overuse), and implementation requirements, including interpretability and computational efficiency [15,17]. This context-specificity underscores the importance of systematic model evaluation for each unique application. Figure 2 summarises the comparative performance of the five primary algorithm families across included studies. Tree-based methods (RF, XGBoost) achieved the highest median AUC–ROC values (0.85–0.95) with consistent performance across sports contexts. NNs showed a slightly narrower confidence interval at high performance levels (median AUC of 0.87–0.93) but with greater between-study variability. SVMs and logistic regression returned lower median AUC–ROC values (0.74 and 0.75, respectively), consistent with their known limitations for high-dimensional tabular data and small samples. XGBoost demonstrated the tightest confidence intervals, reflecting stable performance across the eight studies in which it was the primary method.

Comparative algorithm evaluation reveals context-dependent performance trade-offs. Tree-based methods (RF, XGBoost) achieve strong performance on tabular, structured data (reported AUC range of 0.82–0.95 across included studies; median of 0.85–0.90), with moderate computational cost and inherent interpretability, via feature importance metrics [8,17,26]. NNs demonstrate superiority for high-dimensional time-series and image data (median AUC of 0.87–0.93) but require larger sample sizes (minimum *n* > 500 for stable training), substantial computational resources (graphics processing unit acceleration is mandatory for CNNs), and post hoc explanation methods [7,35]. SVMs optimise performance on small-to-moderate datasets (*n* = 50–200) but scale poorly to high-dimensional feature spaces [13,58]. Algorithm selection should prioritize data structure compatibility, sample size adequacy, and interpretability requirements over isolated accuracy maximization [12,17].

### 3.3. Deep Learning Architectures in the Included Studies

DL techniques have emerged as powerful tools for sports injury prediction, with specific architectures suited to different analytical tasks [6,20]. Convolutional NNs process spatial data through hierarchical feature extraction using convolutional layers and pooling operations [6]. Liang et al. [35] developed a CNN with dual attention mechanisms for magnetic resonance imaging-based ACL-injury detection, achieving 80.6% accuracy and AUC = 0.889 for five-fold cross-validation. Liang et al. [35] developed an explainable CNN for ACL injury risk assessment that incorporated attention mechanisms, highlighting specific movement components influencing predictions and enhancing clinical utility.

ANNs consist of interconnected nodes that model complex, non-linear relationships between injury risk factors [6]. Calderón-Díaz et al. [29] compared 35 ML configurations (including ANN, KNN, SVM, ensembles, and XGBoost) on biomechanical data from 110 male professional soccer players to predict hamstring injury risk; XGBoost achieved the highest precision (78%) and identified maximum hamstring strength and stiffness as the most discriminating predictors. Sanchez et al. [59] reported that combining internal training load (such as session rating of perceived exertion), heart-rate variability, perceptual fatigue, and weekly sleep-disturbance indices identified injury weeks with higher discrimination than did load-only models in endurance athletes. ANNs’ versatility enables the integration of biomechanical parameters, subjective wellness ratings, and other metrics into unified predictive models [29,59]. RNNs are designed for sequential data analysis, with LSTM networks showing particular promise in analysing temporal patterns through their specialized architecture that addresses the vanishing gradient problem inherent in standard RNNs [6,63]. LSTM networks, through memory cells and gating mechanisms, capture long-term dependencies in athlete time-series data relevant for injury prediction [7,63].

More sophisticated approaches leverage architectures that learn joint representations across data modalities. For instance, Dong et al. [64] developed the BioSensor–Transformer, a multimodal architecture integrating inertial measurement units, electromyography, and plantar-pressure data under biomechanical constraints, outperforming state-of-the-art baselines for injury-risk prediction during dynamic movements. Li and Huang [36] demonstrated that combining natural language processing methods with ML for sentiment analysis of athletes’ wellness reports improved injury prediction accuracy by 12% compared to that for physiological data alone.

**Figure 3:**
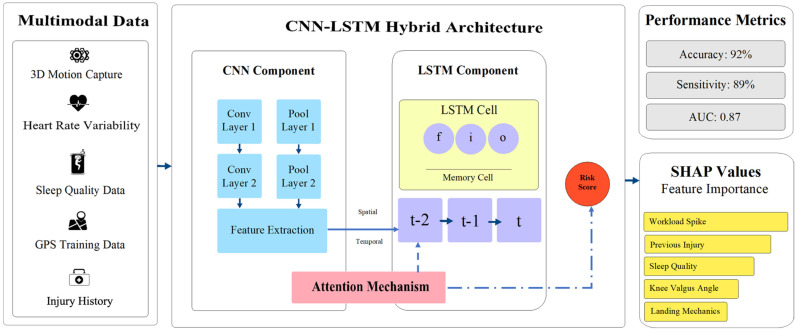
Explainable deep learning architecture for sports injury prediction, illustrating a CNN–LSTM hybrid pipeline. Multimodal inputs (three-dimensional (3D) motion capture, GPS, IMU, heart-rate variability, sleep quality, injury history) are processed through convolutional layers for spatial feature extraction and LSTM cells for temporal dependency modelling. An attention mechanism weights feature importance, and SHAP values quantify individual feature contributions to the final injury risk score, enabling identification of clinically actionable risk factors (e.g., knee valgus angle > 15, sleep quality score < 6/10). AUC = Area Under the Curve; CNN = Convolutional Neural Network; GPS = Global Positioning System; IMU = Inertial Measurement Unit; LSTM = Long Short-Term Memory; SHAP = Shapley Additive Explanations.

Despite superior predictive performance, these models present interpretability challenges given their “black box” nature—where millions of parameters interact in ways that defy straightforward human interpretation [12,65]. Recent studies have focused on enhancing interpretability through attention mechanisms, visualisations, and model-agnostic explanation methods [22,35,60,62]. Liang et al. [35] incorporated attention mechanisms highlighting crucial movement patterns, while other explainable DL methods have been used to support the translation of model outputs into practical interventions. Clinically actionable interpretation requires explicit bridging frameworks. Classification and regression tree algorithms provide threshold values enabling direct intervention targeting. For instance, Kolodziej et al. [33] identified countermovement jump height < 35 cm as an injury risk threshold in youth soccer, directly informing power training prescription. SHAP values quantify feature contributions, translating model outputs into ranked intervention priorities [29,35]. However, most complex NNs lack inherent interpretability, necessitating post hoc explanation methods to convert predictions into clinical reasoning pathways [12,65]. Figure 3 illustrates an explainable DL implementation architecture. The CNN component processes multi-modal input (three-dimensional (3D) motion capture, GPS data, inertial measurement unit sensors) through convolutional layers, extracting spatial features (joint angles, ground reaction forces), as demonstrated by Liang et al. [35]. The LSTM component analyses temporal dependencies across training cycles, capturing fatigue accumulation patterns across training cycles. Attention mechanisms weight feature importance hierarchically, while SHAP values quantify individual feature contributions to injury risk scores [29]. This architecture enables clinicians to identify specific modifiable risk factors (e.g., knee valgus angle > 15, sleep quality score < 6/10) driving predictions, translating black-box outputs into intervention targets [35]. Table 2 provides a comprehensive comparison of NN architectures employed in sports injury prediction. A structured comparison of NN architectures applied to injury prediction is provided in Table 2, highlighting model types, input data, and reported performance metrics. By matching architectural characteristics with appropriate data types and application contexts, researchers can optimise model performance while addressing the inherent complexity of multifactorial injury risk assessment.

Digital systems for managing athlete data have evolved from basic record-keeping platforms to sophisticated monitoring systems incorporating AI-driven analytics. Specialized athlete monitoring platforms now integrate real-time data acquisition, feedback systems, and AI-based injury risk assessments [15,39]. Robertson et al. [37] examined the influence of playing surface on match injury risk in a men’s professional rugby union over six seasons, demonstrating how multi-team monitoring data can be analysed to identify environment-related risk factors. This system utilised a hierarchical decision framework that categorised injury risk factors as modifiable or non-modifiable, directed attention to actionable interventions, and provided sophisticated decision support. AI-informed clinical decision-support systems may enhance prevention through risk stratification and targeted interventions, although effect sizes vary across implementation contexts [15]. The largest prospective evaluation to date, conducted across 14 professional football clubs, reported a 23% overall injury-incidence reduction, with substantial between-site variation, indicating that AI-informed risk stratification supplements rather than replaces comprehensive clinical assessment [62]. These systems translate data into actionable recommendations by integrating multiple data streams through advanced analytical methodologies [15,37].

Learning management systems can integrate with AI-powered systems for injury prediction and prevention, serving as central hubs for data management while delivering personalised training programs. A key advantage of these systems is their capacity for continuous learning and adaptation [16,22]. Rossi et al. [16] developed an online learning framework that updated model parameters weekly based on new training and injury data, maintaining prediction performance across multiple seasons despite changes in team composition and training methods. This adaptive capacity offers more responsive prevention strategies by continuously refining predictive models to adapt to changes in athlete characteristics and training methodologies. Implementation challenges include data quality, standardisation, and user acceptance [4,15]. Nassis et al. [15] addressed user acceptance by developing an athlete-facing mobile application that translated complex AI-generated risk assessments into actionable recommendations using visual risk meters and simplified action plans. This approach resulted in significantly higher adherence to prevention activities compared to that of traditional communication methods. Future development should consider integration with electronic health records and wearable devices, provided that data interoperability and privacy-preserving analytics are ensured. This work will require open standards for data exchange, privacy-preserving analytics, and interoperable system architectures [15,37].

### 3.4. Data Sources and Methodological Approaches

Physiological monitoring (heart-rate variability, biomarkers including creatine kinase and cortisol) indicates recovery status [60]. Evans et al. [38] identified physiological signatures (creatine kinase, cortisol) preceding non-contact lower-limb injuries in professional rugby. Thornton et al. [39] outlined the analytical and visualisation steps required for valid interpretation of athlete-monitoring data in team sports, including methods for determining meaningful change.

Psychological factors are increasingly included in predictive models [46,65]. Li and Huang [36] applied emotional pattern analysis to athletes’ wellness reports, improving prediction accuracy by 12% compared to that of physiological data alone. Johnson et al. [65] demonstrated that environmental and situational variables (playing surface characteristics, ambient temperature, and competitive scheduling) influenced injury susceptibility in collegiate athletes (*n* = 219), increasing model accuracy by 17% through multimodal DL integration. Medical records serve as critical predictors of future injury risk, with enhanced sensitivity for overuse injury prediction when integrated with monitoring data [6]. Methodological approaches involve several key analytical steps. Data preprocessing involves normalization, missing value imputation, and feature scaling. Standardised injury definitions are critical, as definitional variations account for 37% of model performance differences [48]. Feature selection identifies relevant variables, while feature engineering creates new metrics such as acute workload ratios. Kolodziej et al. [33] identified neuromuscular performance parameters as significant injury risk factors in youth football players through systematic feature selection. Data integration combines information from multiple sources into unified datasets through feature-level fusion, decision-level fusion, representation learning, and graph NNs [7,52,53]. Desai [20] demonstrated that stacked modality-specific model ensembles outperform single multimodal models for hamstring injury prediction, while Dong et al. [64] used a transformer-based architecture to fuse multimodal sensor streams under biomechanical constraints, supporting the identification of asymmetrical loading patterns during dynamic movements. Recent work integrating temporal graph encoding with graph NNs has demonstrated improved injury-risk prediction across multiple sports through cross-sport transfer learning [58]. Temporal graph encoding combined with graph NNs has been applied to model dynamic relationships among biomechanical, physiological, and contextual factors, with cross-sport transfer learning frameworks achieving an AUC = 0.826 ± 0.025 across 312 athletes from five sports and maintaining performance under data-scarce conditions [66]. Sequential analysis of well-being and biomechanical streams indicates that subjective and physiological perturbations may precede objective biomechanical compensations, supporting prospective monitoring windows for preventive intervention [59].

Model validation has progressed toward temporal validation, external validation, and dynamic updating approaches. Techniques such as synthetic minority oversampling, class weighting, and focal loss functions were reported to address class imbalance challenges [8,26]. Min et al. [26] and Rommers et al. [8] demonstrated the effective implementation of these techniques in basketball and youth soccer populations, respectively. Visualisation and interpretation techniques enhance clinical utility through explainable ML and DL approaches, highlighting specific factors influencing model predictions, including knee valgus angle during deceleration and asymmetrical ground reaction force distribution during cutting manoeuvres [35]. A systematic categorization of data modalities utilised in comprehensive injury risk assessment is presented in Table 3. The integration challenges identified highlight the necessity for standardised data collection protocols, as mentioned above, in addition to sophisticated fusion methodologies to fully capitalize on the predictive potential of multimodal data streams.

### 3.5. Personalised Injury Prevention Strategies Through AI

#### 3.5.1. From Population-Based to Multidimensional Personalised Prevention

ML and DL methods enable personalised injury prevention beyond population-based programs. Standardised protocols ignore individual risk factor variations [43,68]. ML algorithms process multimodal sensor data to generate individualised risk assessments, enabling targeted prevention strategies based on athlete-specific biomechanical and physiological profiles. The wide variability in athlete responses to standardised prevention programs emphasises the need for personalised approaches that integrate athlete-specific biomechanical, physiological, and psychological profiles [47]. ML- and DL-driven biomechanical analysis identifies athlete-specific movement patterns associated with injury risk. Wang et al. [41] employed a CNN-based system to analyse basketball players’ landing mechanics, informing personalised training programs targeting individual deficits, resulting in a 37% reduction in high-risk landing mechanics compared to standardised programs. As reviewed by Arundale et al. [9], real-time movement quality feedback delivered through markerless motion capture systems with embedded ML algorithms represents a key emerging component of contemporary ACL prevention programmes, with the authors identifying immediate biomechanical feedback during rehabilitation as a direction with strong translational potential for reducing reinjury risk.

Training load management has evolved from threshold-based approaches to optimisation strategies examining individual load–response relationships. Impellizzeri et al. [42,43] developed a reinforcement learning framework that continuously updates individual load–response models, generating personalised training recommendations to minimise injury risk. Van Eetvelde et al. [5] described how computational “digital twins” integrate biomechanical, physiological, and recovery data to predict individual responses to different training scenarios. Real-time feedback systems enable prompt adjustments to training routines. Dallinga et al. [44] demonstrated that an augmented reality system for jump-landing training, coupled with visual feedback based on markerless motion capture and ML analysis, achieved superior adherence and technique improvements compared to those of conventional approaches. Profile-based clustering of athlete-monitoring data has been used to inform individualised recovery and load-management decisions [62]. Miri et al. [45] developed an ML system for ACL reconstruction rehabilitation monitoring that employed multi-task learning to predict reinjury probability and compensation patterns, achieving a 42% reduction in reinjury rates. The probabilistic approach demonstrated by Tabben et al. [54] in the QSL illustrates how even basic ML models, when applied to large longitudinal surveillance datasets, can generate personalised reinjury prevention priorities. The identified injury transition pathways, particularly the groin-to-hamstring sequence (conditional probability of 2.9%) and the within-season hamstring recurrence rate (7.5%), provide a sequenced, evidence-based hierarchy of rehabilitation targets, exemplifying the principle of data-driven individualised prevention at the population level. Psychological factors are increasingly recognized as contributors to injury risk, supporting their integration into monitoring frameworks. Clement et al. [67] created an AI-driven system monitoring linguistic patterns in athletes’ daily wellness reports to identify early psychological distress signs, while Li and Huang [36] demonstrated that sentiment analysis improved prediction accuracy by 12% compared to the results for physiological data alone.

#### 3.5.2. Implementation, Evaluation, and Future Directions

**Figure 4:**
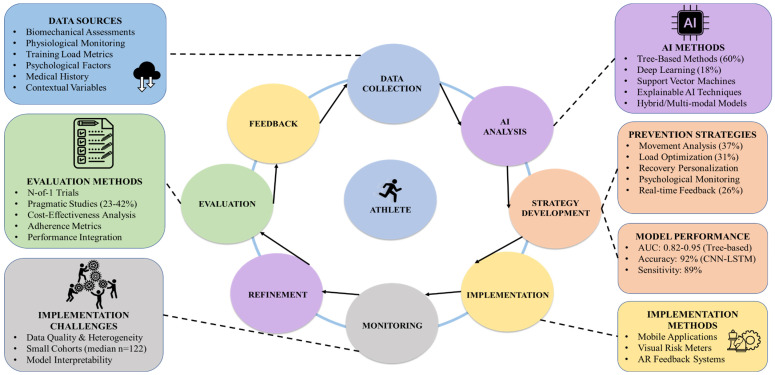
Schematic representation of artificial intelligence (AI)-driven personalised injury prevention approaches. The schematic integrates evidence from included studies to illustrate the full AI-driven prevention cycle, from multimodal data collection through model development and athlete-facing implementation, and maps reported performance metrics and implementation challenges in each stage. AR = Augmented Reality; CNN = Convolutional Neural Network; LSTM = Long Short-Term Memory.

Effective communication of complex risk assessments represents a critical component of successful prevention programs. Nassis et al. [15] developed an athlete-facing mobile application that translated AI-generated risk assessments into actionable recommendations, improving prevention activity adherence. Human-centred design approaches engaging stakeholders throughout development enhanced acceptance of AI-driven load management systems [21,60]. The evaluation of AI-driven prevention strategies employs three complementary approaches. First, single-subject and individualised study designs measuring personalised intervention effects [62] were employed. Second, across included intervention studies, AI-informed prevention strategies were associated with injury reductions ranging from 23% (Souaifi et al. [62], reported as a pooled synthesis across studies implementing integrated AI systems in sports biomechanics) to 42% (Miri et al. [45], multi-task learning for ACL rehabilitation monitoring). These injury reduction estimates should be interpreted with caution: they derive from heterogeneous study designs, populations, and AI systems; the 23% figure represents a synthesis-level estimate from a scoping review [62], while the 42% figure reflects a single-centre prospective intervention [45]; neither estimate derives from a randomised controlled trial, and neither meets the criteria for a clinically deployable system. None of the included studies met the criteria for a clinically deployable system, defined here as requiring external validation on an independent cohort, transparent model reporting, and prospective implementation evidence [4,12]. Third, cost-effectiveness analyses providing economic justification for organizational adoption were included [3]. The historical separation between performance enhancement and injury prevention is counterproductive, as integrated approaches improve both performance and injury outcomes [47]. Malone et al. [47] demonstrated, through an observational cohort study of elite soccer players, that athletes with well-developed physical capacities, specifically high chronic training loads and superior aerobic fitness as measured by the 30–15 Intermittent Fitness Test, maintained lower injury incidence, despite elevated high-speed running demands, indicating that integrated physical preparation frameworks targeting both performance and load tolerance may simultaneously reduce injury risk. The key components of AI-driven personalised prevention, from data collection through implementation and refinement, are shown in Figure 4.

### 3.6. Challenges and Limitations of AI in Sports Injury Prediction

#### 3.6.1. Data-Related Challenges and Privacy Concerns

The included studies identified three primary categories of limitations: (i) data-related challenges (quality, quantity, and privacy); (ii) methodological limitations in model development and validation; and (iii) implementation barriers [4,11]. West et al. [48] demonstrated that variations in injury definitions alone account for up to 37% of performance differences between otherwise identical predictive models. Despite standardisation initiatives such as the “Consensus on Uniform Reporting of Monitoring Data in Team Sports” [39], significant heterogeneity persists across research and practice.

Data quantity and quality constraints operate at multiple levels. Small sample sizes (median of *n* =122) limit generalizability [4,17], although Tabben et al. [54] demonstrate that single-league longitudinal surveillance designs can achieve adequate statistical power (*n* = 1258; 4700 injuries; nine seasons), reducing reliance on class-imbalance correction techniques when injury event volume is sufficient. Nevertheless, low injury incidence (typically 5–15% annual incidence) creates severe class imbalance in most single-season prospective designs, requiring synthetic oversampling or focal loss correction [4,8]. Longitudinal collection across multiple seasons compounds these challenges through temporal non-stationarity (team composition changes, evolving training methodologies) that degrades model stability [39]. Privacy considerations further restrict data pooling; athletes report coercion concerns regarding monitoring consent, with limited understanding of data usage [49]. Federated learning offers partial mitigation by enabling collaborative model training without direct data sharing [62], though computational overhead and communication costs remain as barriers.

#### 3.6.2. Methodological and Implementation Limitations of AI in Sports Injury Prediction

Lack of interpretability in complex models limits clinical utility and reduces stakeholder acceptance [12]. A scoping review with evidence synthesis reported that only 18% of injury prediction models provided sufficient interpretability features to support clinical decision making, despite this being a critical factor for practitioner adoption [12]. Regardless of a growing emphasis on explainable AI approaches, including SHAP values and attention mechanisms, significant challenges remain regarding translating complex outputs into actionable insights. The accuracy–interpretability trade-off represents a fundamental implementation barrier. Models achieving the highest predictive accuracy (deep NNs: AUC = 0.91–0.95) exhibit the lowest interpretability, with only 12% providing clinician-actionable insights without extensive post hoc explanation [12,65]. Conversely, interpretable models (decision trees, logistic regression: AUC = 0.76–0.84) sacrifice 8–15% accuracy but enable direct clinical reasoning [17,29]. Practitioner utilisation and clinical adoption depend on interpretability and actionable outputs, as well as predictive performance, with moderately accurate but interpretable models often achieving superior uptake in real-world settings [15]. This evidence indicates that clinical utility depends more on interpretability and actionable outputs than on raw predictive performance. Models often demonstrate limited generalizability due to variations in training methodologies and athlete characteristics. Transfer learning methodologies show promise for expanding AI-driven prevention access, e.g., adapting models trained on male soccer data for female applications [61]. Most current predictive models identify statistical associations rather than causal relationships, limiting their utility for intervention planning [4,12]. Early work toward mechanistic insight is exemplified by Ayala et al. [31], who employed a decision tree (ADTree)-based ensemble with a synthetic minority oversampling technique (AUC = 0.837) to identify hierarchical relationships between risk factors in hamstring injuries in professional soccer players.

**Figure 5:**
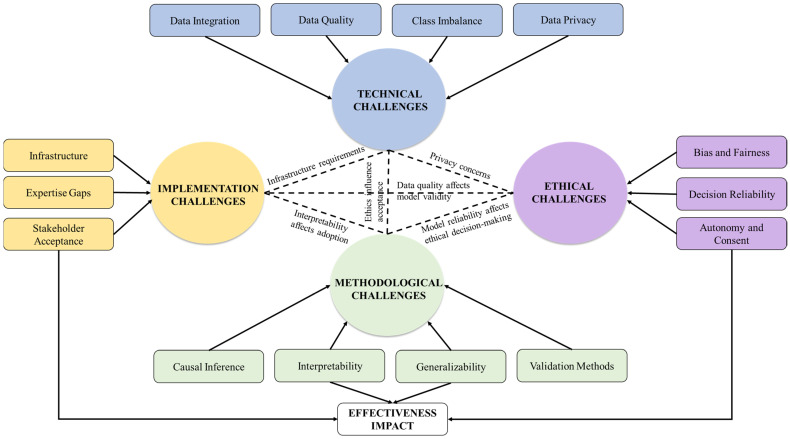
Visual summary of challenges for AI-driven injury prediction and prevention.

Moreover, the effectiveness of AI-driven prevention strategies depends on stakeholder acceptance. Participatory design methodologies for an AI-driven load management system increased utilisation rates compared to that for systems implemented without stakeholder engagement [15]. Additionally, bias introduction from unrepresentative training data may lead to prediction accuracy disparities across demographic groups [28,65]. ML models trained predominantly on male athletes exhibited 23% lower predictive accuracy when applied to female athletes with identical injury types [6], highlighting the critical importance of diverse, representative training datasets [28,65]. Furthermore, limited agreement on evaluation metrics complicates effectiveness comparison across studies [15,50]. Well-calibrated models with moderate discrimination may perform better in practical implementation than do poorly-calibrated models with higher discrimination. Souaifi et al. [62] synthesised evidence from integrated AI systems in sports medicine and reported a 23% reduction in reinjury rates across included implementations, with substantial variation across implementation contexts. Addressing these challenges requires multifaceted strategies. Figure 5 categorizes limitation domains (data integration, technical challenges, methodological constraints, ethical considerations) influencing AI-based injury prediction systems.

## 4. Discussion

### 4.1. Summary of Main Findings

This scoping review mapped the use of AI in sports injury prediction and prevention, identifying a shift from single-variable screening to complex, multi-modal analyses. The 39 included studies showed that tree-based algorithms like RF and XGBoost are common, with DL methods applied to more complex datasets and with probabilistic approaches such as Markov chain models demonstrating utility for sequential reinjury risk quantification in large longitudinal cohorts [54]. A key finding was the integration of diverse data streams, including biomechanical, physiological, and training load metrics, to create more complete athlete profiles. When these AI-driven models informed personalised prevention strategies, studies reported injury reductions between 23% and 42%. Despite these advances, the literature consistently reported methodological challenges, such as small sample sizes, heterogeneous injury definitions, and insufficient model validation. The reported performance metrics and injury reduction figures across included studies must be contextualised within their methodological quality: 62% of studies were rated as having a high or unclear risk of bias using adapted PROBAST criteria (partial or inadequate overall quality), only 13% conducted external validation (5/39), and the median sample size was approximately 122 athletes. Accordingly, current AI models should be regarded as proof-of-concept tools rather than as clinically deployable systems. The implementation challenges identified across included studies are systematically categorised in Table 4.

### 4.2. Methodological and Technological Integration

**Figure 6:**
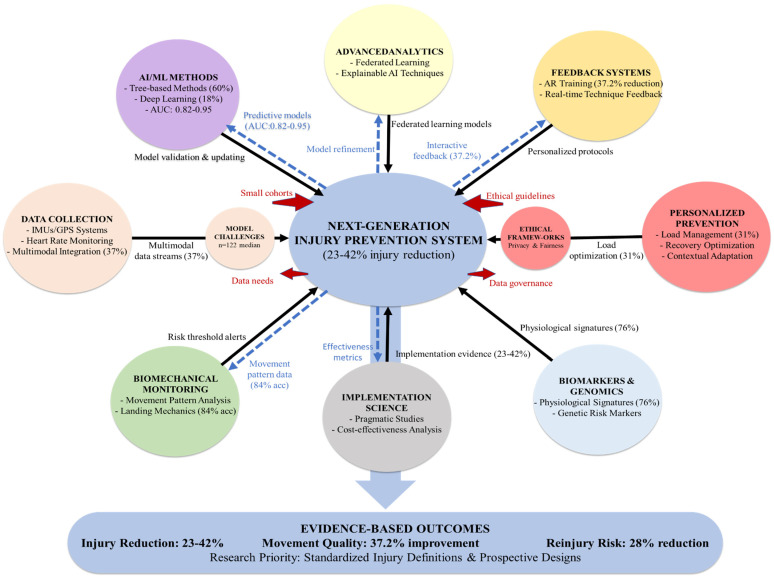
Diagrammatic presentation of technology integration in injury prevention. AI = Artificial Intelligence; AR = Augmented Reality; AUC = Area Under the Curve; GPS = Global Positioning System; IMUs = Inertial Measurement Units.

The findings show a clear progression in the complexity of analytical methods used. The progressive integration of wearable technology, AI analytics, and clinical decision-support tools is illustrated in Figure 6. While earlier computational models in sports science often relied on simpler regression techniques, the studies in this review highlight the now-frequent use of ensemble and DL methods designed to model non-linear relationships within large datasets. Furthermore, the practical application of these models is evolving. The development of clinical decision support systems that translate complex risk outputs into actionable recommendations for practitioners is a significant step [69]. Such systems can help connect model prediction with on-field prevention. However, the effectiveness of these systems is limited by the quality of the input data and the interpretability of the model’s output [4,12].

### 4.3. Limitations and Future Research Directions

A primary limitation identified across the included studies is the lack of methodological standardisation. Future research should prioritize the adoption of consensus-based injury definitions and data-collection protocols, such as those recommended in sports injury surveillance statements [1,39].

Second, many models suffer from limited generalizability [4,12]. A model developed on a specific population, such as elite male soccer players, may not perform well when applied to female athletes or different sports. Validation methodology directly determines clinical applicability. The 15% median accuracy degradation observed during external validation [4,12] indicates that most models overfit training populations. Temporal validation should constitute minimum standards, with external validation across different teams, sports, or competitive levels as mandatory before clinical implementation [12]. Pre-registration of validation protocols and prospective evaluation designs would reduce reporting bias and selective outcome reporting [56,57]. To address this, future work should include robust external validation across diverse populations. There is a particular need for more research focused on female athletes, who are underrepresented in the current literature [6]. Priorities and key technologies for each future research direction are detailed in Table 5.

Finally, improving model interpretability is essential for clinical adoption [12]. The “black box” nature of some complex models is a major barrier to trust and implementation [65]. Future studies should continue to integrate explainable AI techniques that clarify which factors are driving a prediction, making the outputs more transparent and useful for coaches and medical staff. Rigorous implementation studies are also needed to evaluate how these AI systems perform in real-world settings and to determine the organizational factors that influence their adoption and success [10].

Three search-process limitations warrant acknowledgement. First, restricting eligibility to publications in English, French, and German may have introduced language bias; relevant studies published in other languages cannot be retrospectively retrieved within the scope of this review. Second, the Google Scholar search was bounded to the first 200 records ranked by relevance per query string and conducted in a logged-out browser session to minimise personalisation effects, as documented in the Supplementary Materials; this ceiling was applied prospectively and is reproducible, though it does not guarantee exhaustive retrieval from that source. Third, one minor deviation from the registered protocol (OSF.IO/4nd95) was made: a post-submission targeted review of Biology of Sport 2026 early-access publications identified one additional eligible study [54] meeting all inclusion criteria, which was incorporated following consensus review by W.D. and N.J.; all other protocol elements were adhered to as registered.

## 5. Conclusions

AI methodologies demonstrate increasing sophistication in analysing multimodal datasets for sports injury prediction applications. Evidence supports injury reduction efficacy when AI-informed models guide personalised prevention strategies. However, significant methodological barriers persist, including data heterogeneity, inadequate validation protocols, and limited model interpretability. Addressing these fundamental limitations through standardised approaches, comprehensive validation frameworks, and enhanced model transparency represents a critical requirement for translating research advances into effective clinical implementation. Notably, the largest longitudinal cohort in the reviewed corpus demonstrates that probabilistic ML models applied to adequately powered surveillance datasets can yield interpretable, actionable reinjury risk estimates, providing a scalable template for future multi-season injury prevention research.

## Figures and Tables

**Table 1:** Evolution and applications of artificial intelligence (AI) methodologies in sports injury prediction.

Evolutionary Period	Methodological Characteristics	Representative Studies and Outcomes	Methodological Limitations
2018–2020: Foundational approaches	Logistic regression Decision trees Support vector machines Simple neural networks Limited feature sets Primarily univariate analyses Retrospective study designs	Rossi et al. [18]: GPS training data analysis in soccer (AUC: 0.70). Claudino et al. [11]: Systematic review identifying methodological limitations in early applications. Cust et al. [22]: Systematic review of ML and deep learning for sport-specific movement recognition, identifying ensemble methods achieving AUC: 0.79. Thornton et al. [39]: Development of training load monitoring frameworks. Karnuta et al. [28]: Ensemble ML applied to 13,982 player-years from major league baseball (top three-ensemble AUC 0.76 position players; 0.65 pitchers). Ayala et al. [31]: Decision-tree (ADTree) base classifier with SMOTE oversampling and boosting ensembles for hamstring strain injury prediction in professional soccer (AUC 0.837; sensitivity 77.8%; specificity 83.8%).	Limited feature engineering Inadequate handling of class imbalance Insufficient validation procedures Incomplete reporting of model parameters Limited assessment of calibration
2021–2022: Intermediate refinement	Random forests Gradient boosting XGBoost Basic deep learning Integration of multimodal data Improved feature selection Emergence of prospective designs	Cust et al. [22]: Ensemble methods for movement recognition in injury risk assessment (AUC: 0.79). Luu et al. [27]: XGBoost for next-season hockey injuries (AUC: 0.948). Calderón-Díaz et al. [29]: KNN and ANN for hamstring injury prevention models.	Moderate model interpretability Limited time-series analysis capabilities Focus on statistical association rather than causation Variable reporting of hyperparameter tuning Inconsistent validation protocols
2023–2026: Advanced integration	Sophisticated deep learning (CNN, RNN, LSTM) Hybrid/ensemble models Explainable AI approaches Time-series analysis Real-time prediction capabilities Federated learning implementations	Wang et al. [41]: 3D convolutional neural network for basketball technique action recognition supporting movement-quality screening. Mendiguchia et al. [60]: Explainable AI identifying biomechanical injury risk factors. Tabben et al. [54]: Markov chain probabilistic model applied to nine competitive seasons of injury data from 1258 professional football players in the Qatar Stars League; 4700 injuries recorded, with 34% identified as subsequent injuries; within-season hamstring recurrence probability 7.5% (±1.3%); groin-to-hamstring injury transition probability 2.9% (±0.82%); largest longitudinal dataset in the corpus; exemplifies interpretable, low-complexity probabilistic ML for clinical reinjury risk stratification. Liang et al. [35]: CNN with two attention-mechanism modules for ACL-injury detection on MRI (AUC = 0.889; accuracy 80.6%). Li and Huang [36]: Comprehensive survey; NLP + ML hybrid (sentiment analysis of wellness reports as sub-finding) using sentiment analysis (12% improvement over the use of physiological data alone).	Complex implementation requirements Significant computational demands Clinical interpretability challenges Limited external validation Ethical considerations regarding algorithm transparency Inconsistent stakeholder engagement

ACL = Anterior Cruciate Ligament; ANN = Artificial Neural Network; AUC = Area Under the Curve; CNN = Convolutional Neural Network; GPS = Global Positioning System; KNN = K-Nearest Neighbours; LSTM = Long Short-Term Memory; ML = Machine Learning; MRI = Magnetic Resonance Imaging; NLP = Natural Language Processing; RNN = Recurrent Neural Network; SMOTE = Synthetic Minority Oversampling Technique; XGBoost = Extreme Gradient Boosting.

**Table 2:** Neural network architectures and data integration for sports injury prediction.

Neural Network Type	Key Capabilities	Data Requirements	Example Applications	Representative Studies
Convolutional neural networks (CNNs)	Image and video analysis Spatial pattern recognition Automated feature extraction Hierarchical representation learning	Video footage Motion-capture data Biomechanical heat maps Visual movement assessments Structured spatial data	Movement quality assessment Technique analysis Posture evaluation Landing mechanics assessment Cutting-manoeuvre analysis	Wang et al. [41]: 3D CNN-based recognition of high-risk landing and cutting actions. Liang et al. [35]: CNN with attention mechanisms for ACL injury risk assessment.
Artificial neural networks (ANNs)	Complex non-linear modelling Multivariate analysis Pattern recognition Categorical variable handling	Structured numerical data Categorical variables Mixed-data formats Pre-processed feature sets	General injury risk assessment Multifactorial analysis Classification problems Risk stratification	Calderón-Díaz et al. [29]: Comparative evaluation of 35 ML configurations including ANN; XGBoost selected as best model (precision 78%) for *n* = 110 professional soccer players. Sanchez et al. [59]: Integration of heart-rate variability and sleep-disturbance metrics for injury monitoring in endurance athletes.
Recurrent neural networks (RNNs)	Sequential data analysis Temporal pattern recognition Variable-length input processing Time-series modelling	Time-series data Longitudinal measurements Sequential patterns Temporal relationships	Training load progression analysis Fatigue monitoring Adaptation tracking Temporal risk assessment	Al-Selwi et al. [63]: Systematic review of RNN-LSTM applications and modelling techniques.
Long short-term memory (LSTM)	Long-term dependency modelling Variable time interval handling Memory cell integration Gradient vanishing problem mitigation	Extended time-series Irregularly sampled data Long-term sequence data Temporal dependencies	Chronic overuse injury prediction Long-term load monitoring Adaptation tracking Sequential risk assessment	Al-Selwi et al. [63]: Theoretical foundations and applications of LSTM architectures.
Hybrid/Multimodal architectures	Cross-modal integration Complex system modelling Multi-source fusion Attention-weighted learning	Multiple data streams Heterogeneous data types Multi-sensor inputs Diverse modalities	Comprehensive risk profiling Environment–athlete interaction Multi-factor analysis Contextual understanding	Dong et al. [64]: Multimodal BioSensor–Transformer integrating IMU, EMG, and plantar-pressure data with biomechanical constraints. Meng & Qiao [30]: Dual-feature fusion neural network for sports injury estimation.

ACL = Anterior Cruciate Ligament; ANN = Artificial Neural Network; XGBoost = Extreme Gradient Boosting; 3D = Three-Dimensional.

**Table 3:** Data modalities and integration approaches for comprehensive injury risk assessment.

Data Category	Key Parameters	Collection Methods	Integration Approaches	Implementation Challenges	Representative Studies
Biomechanical	Joint angles Ground reaction forces Movement patterns Biomechanical asymmetries Neuromuscular control Technique parameters	3D motion capture Force plates Inertial measurement units Video analysis Markerless tracking Computer vision	CNN processing Pose estimation Time-series analysis Biomechanical modelling Movement pattern recognition	Ecological validity Laboratory vs. field measurements Equipment cost and accessibility Data synchronization Processing complexity	Liang et al. [35]: Analysis of 3D motion capture for jump-landing mechanics. Wang et al. [41]: Computer-vision analysis of basketball landing mechanics via 3D CNN.
Physiological & Biological	Heart-rate variability Sleep quality metrics Biomarkers (CK, cortisol) Hormonal profiles Inflammatory markers Metabolic indicators	Wearable monitors Point-of-care testing Laboratory assays Sleep tracking Genetic screening Continuous monitoring	Multivariate time-series models Sequential pattern mining Biomarker embedding Physiological modelling Temporal relationship analysis	Inter-individual variability Measurement consistency Environmental influences Circadian variations Interpretation complexity	Li et al. [36,51]: Pre-sleep heart-rate variability as a predictor of chronic insomnia and sleep continuity in national-level athletes. Evans et al. [38]: Biomarker analysis for rugby soft tissue injuries (76% accuracy). Evan et al. [38]: Analysis of physiological signatures preceding injuries.
Training load & Recovery	External load metrics Internal load measures Acute: Chronic Workload Ratio Neuromuscular performance Recovery metrics Adaptation indicators	GPS/LPS systems Heart-rate monitoring Subjective ratings Jump tests Wellness questionnaires Mobile applications	Temporal pattern recognition LSTM networks Dynamic weighting Load–response modelling Adaptation tracking Individual baseline comparisons	Individual response variations Context-specific interpretations Methodological inconsistencies Integration with competition data Periodization influences	Thornton et al. [39]: Methodological framework for analysis and visualisation of athlete-monitoring data. Rossi et al. [18]: GPS data for forecasting wellness in soccer. Impellizzeri et al. [42]: Load–response modelling frameworks. Malone et al. [47]: High-speed running as injury risk factor.
Psychological & Contextual	Stress levels Mood fluctuations Environmental conditions Competition schedule Team dynamics Travel demands	Validated questionnaires Electronic diaries Team records Meteorological data Schedule analysis Natural language processing	Natural language processing Contextual embedding Multimodal fusion Environmental modelling Schedule analysis Psychological profiling	Subjective reporting biases Cultural and individual variations Limited standardisation Integration with physiological data Privacy considerations	Johnson et al. [65]: Environmental and situational variables (17.3% increase in model accuracy). Li and Huang [36]: Sentiment analysis of wellness reports (12% accuracy improvement). Clement et al. [67]: Linguistic pattern monitoring.
Medical records & History	Previous injuries Rehabilitation outcomes Medical conditions Treatment responses Surgical history Medication profiles	Electronic health records Injury databases Clinical assessments Return-to-play evaluations Rehabilitation tracking	Medical concept embedding Temporal pattern analysis Risk factor modelling Treatment response prediction Reinjury risk assessment	Data accessibility Privacy regulations Standardisation limitations Inter-practitioner variability Documentation inconsistencies	Mendiguchia et al. [60]: Anterior pelvic tilt as key factor in hamstring strain. Miri et al. [45]: Post-ACL reconstruction risk assessment.

ACL = Anterior Cruciate Ligament; CK = Creatine Kinase; CNN = Convolutional Neural Network; GPS/LPS = Global Positioning System/Local Positioning System; LSTM = Long Short-Term Memory; 3D = Three-Dimensional.

**Table 4:** Implementation challenges in artificial intelligence (AI)-based injury prediction and prevention.

Challenge Domain	Current Limitations	Methodological Implications	Emerging Solutions	Research Priorities	Key References
Data quality & heterogeneity	Inconsistent injury definitions Variable measurement protocols Sensor calibration differences Subjective reporting biases Heterogeneous data formats	Reduced model accuracy Limited generalizability Restricted cross-study comparison Misleading performance metrics Variation in injury classification	Standardised reporting frameworks Multi-source verification protocols Calibration harmonization Consensus-based definitions Common data elements	Development of consensus injury definitions Validation of measurement equivalence Establishment of minimum reporting standards Cross-platform calibration approaches Data quality assessment frameworks	West et al. [48]: Injury definition variation accounting for 37% of model performance differences. Bullock et al. [4]: Systematic review of methodological limitations. Leckey et al. [12]: Machine learning approaches to injury prediction.
Sample size & statistical power	Limited sample sizes (median of *n* = 122) Class imbalance (rare injury events) Insufficient injury occurrences Limited longitudinal tracking Fragmented populations	Overfitting to training data Poor generalization capacity Limited statistical power Unstable model performance Insufficient validation	Multi-centre collaborative databases Synthetic minority oversampling Class weighting approaches Transfer-learning techniques Data augmentation methods Long-duration single-league longitudinal surveillance designs	Development of sample-size guidelines Standardisation of validation protocols Implementation of robust evaluation frameworks Collaborative data initiatives Privacy-preserving analytics	Min et al. [26]: Class imbalance handling techniques. Lee Dow et al. [32]: Limitations of small cohort studies. Rommers et al. [8]: Population-specific recalibration needs. Kolodziej et al. [33]: Feature selection for limited samples. Tabben et al. [54]: Nine-season longitudinal cohort (*n* = 1258) demonstrating that sufficient injury event volume (4700 injuries) in single-league surveillance designs reduces class imbalance constraints for probabilistic ML models.
Model interpretability	“Black box” model limitations Limited causal understanding Complex hyperparameter tuning Obscured decision-making processes Stakeholder comprehension barriers	Reduced clinical trust Limited intervention guidance Implementation resistance Ethical concerns Regulatory challenges	Explainable AI techniques (SHAP values) Attention mechanisms Feature importance visualization Model-agnostic explanation methods Hybrid knowledge-based models	Development of interpretability standards Clinical integration frameworks Stakeholder engagement methodologies Causal inference integration User-centred design approaches	Liang et al. [35]: Attention mechanisms for model interpretation. Mendiguchia et al. [60]: Explainable AI for hamstring injury factors. Leckey et al. [12]: Only 18% of injury prediction models with sufficient interpretability. Ayala et al. [31]: Causal discovery algorithms.
Implementation & adoption	Context-dependent performance Limited stakeholder engagement Technical implementation barriers Organizational resistance Resource constraints	Variable real-world effectiveness Suboptimal adoption patterns Limited clinical impact Knowledge translation gaps Performance-implementation paradox	User-centred design approaches Implementation science integration Participatory development Simplified interfaces Contextualized deployment	Clinical integration frameworks Implementation outcome evaluation Knowledge-translation strategies Organizational readiness assessment Cost-effectiveness analyses	Nassis et al. [15]: Participatory design approaches. Souaifi et al. [62]: Synthesis of integrated AI implementations reporting 23% reinjury-rate reduction across included studies. Robertson et al. [37]: Federated approaches for privacy.

SHAP = SHapley Additive exPlanations.

**Table 5:** Future research directions for artificial intelligence (AI)-based injury prediction and prevention.

Future Research Directions	Key Technologies	Application Areas	Expected Impact	Development Priorities	References
Advanced model development	Hybrid mechanistic–statistical models Physics-informed neural networks Causal inference integration Knowledge-guided deep learning Ensemble meta-learning	Biomechanical injury risk assessment Load–response modelling Tissue adaptation monitoring Movement-quality evaluation Injury-mechanism understanding Sequential reinjury risk modelling using probabilistic state-transition approaches	Enhanced model interpretability Improved generalizability Better causal understanding More efficient knowledge utilization Reduced data requirements	Integration of domain knowledge Mechanistic constraint implementation Causal structure modelling Mechanistic–statistical frameworks Explainable AI architectures	Johnson et al. [65]: On-field workload monitoring via deep learning. Mendiguchia et al. [60]: Identification of biomechanical risk factors. Tabben et al. [54]: Markov chain probabilistic modelling of sequential injury transitions in 1258 QSL football players; actionable reinjury probability estimates across nine seasons.
Privacy-preserving analytics	Federated learning Differential privacy techniques Synthetic data generation Edge computing implementations Privacy-by-design frameworks	Cross-institutional collaboration Competitive team environments Multi-site research initiatives Global data registries Longitudinal monitoring	Enhanced data-sharing capabilities Expanded collaborative model development Improved model generalizability Protection of proprietary information Ethical data utilization	Decentralized learning frameworks Privacy-performance optimisation Secure multi-party computation Distributed validation techniques Regulatory compliance frameworks	Robertson et al. [37]: Multi-team prospective monitoring analysis across professional rugby clubs, demonstrating collaborative multi-site data integration for environment-related injury risk factor identification. Li et al. [36]: Privacy and data rights considerations in sports monitoring contexts. Kolodziej et al. [33]: Feature selection and transfer learning across youth football populations.
Adaptive intervention design	Reinforcement learning Multi-objective optimisation Simulation-based training Digital twin modelling Adaptive feedback systems	Personalised load management Individualised prevention programs Rehabilitation optimisation Return-to-play decision support Performance–injury balance	Continuously evolving prevention strategies Context-specific intervention optimisation Individual response adaptation Real-time intervention adjustment Performance–injury risk balance	Multi-objective reward function design Simulation environment development Safety constraint implementation Individual response modelling Real-world validation frameworks	Impellizzeri et al. [42]: Multi-objective optimisation framework. Miri et al. [45]: Multi-task learning for rehabilitation monitoring. Wang et al. [41]: Personalised training program targeting. Dallinga et al. [44]: Augmented reality for jump-landing training.
Natural language interfaces	Large language models (GPT-4, Claude) Sport-specific model fine-tuning Multimodal interaction systems Context-aware dialogue Simplified visualization	Knowledge translation Risk communication Athlete education Intervention guidance Implementation support	Enhanced accessibility of complex models Improved stakeholder comprehension Increased implementation adherence Simplified decision support Democratized analytical tools	Domain-specific model adaptation Clinical validation of outputs User experience optimisation Information accuracy verification Implementation effectiveness assessment	Dergaa et al. [70]: Evaluation of GPT-4 for exercise prescription. Ben Saad et al. [71]: Impact of AI chatbots on cognitive health. Hamdaoui et al. [72]: Integration implications in sports science. Dergaa et al. [73]: Limitations in mental health assessment.
Democratised applications	Simplified monitoring technologies Mobile application integration Cloud-based analytics Low-cost sensor systems Accessible assessment tools	Youth sports injury prevention Recreational athlete monitoring Community sport applications Public health integration Educational implementation	Expanded access beyond elite contexts Public health impact Career longevity enhancement Knowledge translation to diverse settings Preventive culture development	User-centered design for diverse populations Validation across implementation contexts Simplified data collection protocols Resource-appropriate adaptations Implementation science integration	Stampfler et al. [74]: Smartphone-based activity recognition. Nassis et al. [15]: Mobile applications for risk assessment. Rommers et al. [8]: Machine learning in youth football.

GPT-4 = Generative Pre-Trained Transformer 4; QSL: Qatar Stars League.

## Data Availability

The original contributions presented in this study are included in the article/Supplementary Materials. Further inquiries can be directed to the corresponding authors.

## Supplementary Materials

The following supporting information can be downloaded at: https://www.mdpi.com/article/10.3390/bioengineering13060692/s1, Table S1: PRISMA 2020 checklist with Item-by-Item cross-references; Table S2: Characteristics of Included Studies on Artificial Intelligence (AI) Applications for Sports Injury Prediction and Prevention (*n* = 38); Table S3: Structured methodological quality assessment of included studies based on adapted PROBAST and TRIPOD frameworks (*n* = 38); Supplementary Material: Full Boolean Search Strings and Database-Specific Syntax.
