# Supplementary material for: Application of Artificial Intelligence for Predicting Sports Injuries and Customizing Personalized Prevention Strategies: A Scoping Review
Source: Bioengineering (Basel). 2026 Jun 17;13(6):692. doi: 10.3390/bioengineering13060692 (PMC13296066; doi:10.3390/bioengineering13060692)
Supplement: Supplementary file 1 [file bioengineering-13-00692-s001.zip › bioengineering-4346431-supplementary.pdf]

bioengineering MDPI

SUPPLEMENTARY MATERIALS

Supplementary Table S1. PRISMA 2020 Checklist with Item-by-Item Cross-References.

Manuscript: Application of Artificial Intelligence for Predicting Sports Injuries and Customizing Personalised Prevention Strategies: A Scoping Review (bioengineering-4346431)

Reporting framework: PRISMA 2020 (**Page et al., 2021**), complemented by the PRISMA-ScR extension (**Tricco et al., 2018**) given the scoping-review design.

Section / Topic	Item No.	Checklist Item	Location in Manuscript (Section; Page)	Reported (Yes / No / NA)
**TITLE**				
Title	1	Identify the report as a systematic review.	Title page (p. 1); manuscript identified as "A Scoping Review"	Yes
**ABSTRACT**				
Abstract	2	See the PRISMA 2020 for Abstracts checklist.	Abstract (p. 2); structured under Background, Objective, Methods, Results, Conclusions	Yes
**INTRODUCTION**				
Rationale	3	Describe the rationale for the review in the context of existing knowledge.	Section 1 Introduction (pp. 4–5); includes definitions of "personalised prevention strategies" and "translational barriers" inserted at first occurrence	Yes
Objectives	4	Provide an explicit statement of the objective(s) or question(s) the review addresses.	Section 1 Introduction, final paragraph (p. 5): overarching research question inserted; three aims stated	Yes
**METHODS**				
Eligibility criteria	5	Specify the inclusion and exclusion criteria for the review and how studies were grouped for the syntheses.	Section 2.3 Eligibility criteria (p. 5); multi-algorithm handling rule added at end of section	Yes
Information sources	6	Specify all databases, registers, websites, organisations, reference lists, and other sources searched or consulted to identify studies. Specify the date when each source was last searched or consulted.	Section 2.2 Search strategy (p. 5); five databases (PubMed, Web of Science, IEEE Xplore, Scopus, Google Scholar); date through February 2026; grey literature exclusion clarified	Yes
Search strategy	7	Present the full search strategies for all databases, registers and	Section 2.2 Search strategy (p. 5); full Boolean strings with database-specific syntax, MeSH term	Yes

		websites, including any filters and limits used.	lists, and Google Scholar sampling protocol (first 200 records ranked by relevance per Haddaway et al., 2015) provided in Supplementary Material	
Selection process	8	Specify the methods used to decide whether a study met the inclusion criteria, including how many reviewers screened each record and each report retrieved, whether they worked independently, and, if applicable, details of automation tools used in the process.	Section 2.4 Study selection (p. 5); two independent reviewers (WD, NJ); discrepancies ($n = 14$) resolved by structured consensus re-examination of full text against eligibility criteria (§2.3); inter-rater agreement $\kappa = 0.87$ [26]; third-reviewer adjudication not required	Yes
Data collection process	9	Specify the methods used to collect data from reports, including how many reviewers collected data from each report, whether they worked independently, any processes for obtaining or confirming data from study investigators, and, if applicable, details of automation tools used in the process.	Section 2.5 Data extraction and charting (p. 6); extraction form piloted on ten randomly selected eligible studies prior to full deployment [24]; discrepancies <5% resolved by consensus; corresponding authors contacted when methodological details were insufficiently reported	Yes
Data items	10a	List and define all outcomes for which data were sought. Specify whether all results that were compatible with each outcome domain in each study were sought (e.g., for all measures, time points, analyses), and, if not, the methods used to decide which results to collect.	Section 2.5 Data extraction and charting (p. 6, items i–vii)	Yes
	10b	List and define all other variables for which data were sought (e.g., participant and intervention characteristics, funding sources). Describe any assumptions made about any missing or unclear information.	Section 2.5 Data extraction and charting (p. 6, items i–vii)	Yes
Study risk of bias assessment	11	Specify the methods used to assess risk of bias in the included studies, including details of the	Section 2.6 Quality assessment (p. 6); adapted PROBAST (Moons et al., 2019) selected for risk-of-bias assessment across participant, predictor, outcome,	Yes (adapted; non-

		tool(s) used, how many reviewers assessed each study and whether they worked independently, and, if applicable, details of automation tools used in the process.	and analysis domains; TRIPOD (Collins et al., 2015) applied for reporting completeness; aggregate results tabulated in Supplementary Table S3; non-mandatory for scoping reviews per Tricco et al. (2018)	mandatory for scoping reviews per Tricco et al., 2018)
Effect measures	12	Specify for each outcome the effect measure(s) (e.g., risk ratio, mean difference) used in the synthesis or presentation of results.	NA — narrative scoping synthesis without quantitative pooling	NA
Synthesis methods	13a	Describe the processes used to decide which studies were eligible for each synthesis (e.g., tabulating the study intervention characteristics and comparing against the planned groups for each synthesis).	Section 2.5 Data extraction and charting; Section 2.6 Quality assessment (p. 6)	Yes
	13b	Describe any methods required to prepare the data for presentation or synthesis, such as handling of missing summary statistics, or data conversions.	Section 2.5 Data extraction and charting (p. 6); author contact when required	Yes
	13c	Describe any methods used to tabulate or visually display results of individual studies and syntheses.	Section 3 Results; Tables 1–5; Figures 2–6 (pp. 6–18)	Yes
	13d	Describe any methods used to synthesize results and provide a rationale for the choice(s). If meta-analysis was performed, describe the model(s), method(s) to identify the presence and extent of statistical heterogeneity, and software package(s) used.	Section 3 Results; narrative synthesis with thematic categorisation	Yes (qualitative synthesis only)
	13e	Describe any methods used to explore possible causes of heterogeneity among study results (e.g., subgroup analysis, meta-regression).	NA — no quantitative pooling performed	NA
	13f	Describe any sensitivity analyses conducted to assess robustness of the synthesised results.	NA — qualitative synthesis	NA

Reporting bias assessment	14	Describe any methods used to assess risk of bias due to missing results in a synthesis (arising from reporting biases).	NA — non-mandatory for scoping reviews per Tricco et al. (2018)	NA
Certainty assessment	15	Describe any methods used to assess certainty (or confidence) in the body of evidence for an outcome.	NA — non-mandatory for scoping reviews per Tricco et al. (2018)	NA
**RESULTS**				
Study selection	16a	Describe the results of the search and selection process, from the number of records identified in the search to the number of studies included in the review, ideally using a flow diagram.	Figure 1 (PRISMA 2020 flow diagram, updated to n=39); Section 2.4 Study selection (p. 5); one post-submission inclusion (Tabben et al. [54]) noted as minor protocol deviation in Section 4.3	Yes
	16b	Cite studies that might appear to meet the inclusion criteria, but which were excluded, and explain why they were excluded.	Figure 1 exclusion reasons (n = 20 at full-text: insufficient methodological details, n = 8; no injury-prediction focus, n = 7; non-sporting populations, n = 5)	Yes
Study characteristics	17	Cite each included study and present its characteristics.	Supplementary Table S2	Yes
Risk of bias in studies	18	Present assessments of risk of bias for each included study.	Section 2.6 Quality assessment (p. 6); narrative appraisal embedded in Section 3.7 (pp. 12–13); structured summary in Supplementary Table S3	Yes (adapted)
Results of individual studies	19	For all outcomes, present, for each study: (a) summary statistics for each group (where appropriate) and (b) an effect estimate and its precision (e.g., confidence/credible interval), ideally using structured tables or plots.	Supplementary Table S2 (performance metrics column); Section 3 narrative	Yes
Results of syntheses	20a	For each synthesis, briefly summarise the characteristics and risk of bias among contributing studies.	Section 3.1–Section 3.7 (pp. 6–18)	Yes
	20b	Present results of all statistical syntheses conducted. If meta-analysis was done, present for each the summary estimate and its precision (e.g.,	NA — narrative synthesis only	NA

		confidence/credible interval) and measures of statistical heterogeneity. If comparing groups, describe the direction of the effect.		
	20c	Present results of all investigations of possible causes of heterogeneity among study results.	NA	NA
	20d	Present results of all sensitivity analyses conducted to assess the robustness of the synthesised results.	NA	NA
Reporting biases	21	Present assessments of risk of bias due to missing results (arising from reporting biases) for each synthesis assessed.	NA — non-mandatory for scoping reviews	NA
Certainty of evidence	22	Present assessments of certainty (or confidence) in the body of evidence for each outcome assessed.	NA — non-mandatory for scoping reviews	NA
**DISCUSSION**				
Discussion	23a	Provide a general interpretation of the results in the context of other evidence.	Section 4.1 Summary of main findings (p. 13); Section 4.2 Methodological and technological integration (p. 13)	Yes
	23b	Discuss any limitations of the evidence included in the review.	Section 3.7 Challenges and limitations (pp. 12–13); Section 4.3 Limitations and future research directions (pp. 13–14)	Yes
	23c	Discuss any limitations of the review processes used.	Section 4.3 (pp. 13–14); includes language bias, Google Scholar boundary, and protocol-deviation statement	Yes
	23d	Discuss implications of the results for practice, policy, and future research.	Section 4.3 (pp. 13–14); Section 5 Conclusions (p. 14); Tables 4 and 5	Yes
**OTHER INFORMATION**				
Registration and protocol	24a	Provide registration information for the review, including the register name and registration number, or state that the review was not registered.	Section 2.1 Protocol and registration (p. 5): Open Science Framework; OSF.IO/4nd95; https://osf.io/4nd95	Yes

	24b	Indicate where the review protocol can be accessed, or state that a protocol was not prepared.	Section 2.1 (p. 5); protocol available at https://osf.io/4nd95	Yes
	24c	Describe and explain any amendments to information provided at registration or in the protocol.	One minor amendment: post-submission identification and inclusion of one additional eligible study (Tabben et al. **[54];** Biology of Sport 2026;43(1):489–498; doi:10.5114/biolsport.2026.152345) following targeted early-access review; inclusion confirmed by consensus (WD, NJ) against eligibility criteria in section 2.3.	Yes
Support	25	Describe sources of financial or non-financial support for the review, and the role of the funders or sponsors in the review.	Declarations — Funding: "There is no funding to declare."	Yes
Competing interests	26	Declare any competing interests of review authors.	Declarations — Conflict of interest	Yes
Availability of data, code and other materials	27	Report which of the following are publicly available and where they can be found: template data collection forms; data extracted from included studies; data used for all analyses; analytic code; any other materials used in the review.	Section 2.1 (revised); Supplementary Table S2 contains extracted data; protocol and data-extraction form available on request to the corresponding author and via OSF registration (https://osf.io/4nd95)	Yes

Abbreviations. NA = not applicable; **OSF** = Open Science Framework; **PRISMA** = Preferred Reporting Items for Systematic Reviews and Meta-Analyses; **PRISMA-ScR** = PRISMA extension for Scoping Reviews; **PROBAST** = Prediction Model Risk of Bias Assessment Tool; **TRIPOD** = Transparent Reporting of a multivariable prediction model for Individual Prognosis or Diagnosis.

Notes. Items 12, 13e, 13f, 14, 15, 20b, 20c, 20d, 21, and 22 are designated "Not Applicable" because they pertain to quantitative synthesis (meta-analysis), risk-of-bias-in-synthesis assessment, and certainty-of-evidence grading, which are reporting elements specific to systematic reviews of intervention or prognostic effects and are not required for scoping reviews per the PRISMA-ScR extension (Tricco et al., 2018). Item 11 (risk of bias in individual studies) was nonetheless addressed using adapted PROBAST and TRIPOD frameworks to enhance methodological transparency. A structured summary of the quality assessment results across all 38 included studies, including validation strategy, external validation status, performance metric reporting, and overall quality rating, is provided in Supplementary Table S3.

References cited in this checklist.

Page, M. J., et al. (2021). The PRISMA 2020 statement: An updated guideline for reporting systematic reviews. BMJ, 372, Article n71. https://doi.org/10.1136/bmj.n71

Tricco, A. C., et al. (2018). PRISMA extension for scoping reviews (PRISMA-ScR): Checklist and explanation. Annals of Internal Medicine, 169(7), 467–473. https://doi.org/10.7326/M18-0850

Moons, K. G. M., et al. PROBAST: A tool to assess risk of bias and applicability of prediction model studies: Explanation and elaboration. Annals of Internal Medicine, 170(1), W1–W33. https://doi.org/10.7326/M18-1377

Collins, G. S., et al. (2015). Transparent reporting of a multivariable prediction model for individual prognosis or diagnosis (TRIPOD): The TRIPOD statement. BMC Medicine, 13, Article 1. https://doi.org/10.1186/s12916-014-0241-z

Haddaway, N. R., et al. (2015). The role of Google Scholar in evidence reviews and its applicability to grey literature searching. PLOS ONE, 10(9), Article e0138237. https://doi.org/10.1371/journal.pone.0138237

Supplementary Table S2. Characteristics of Included Studies on Artificial Intelligence (AI) Applications for Sports Injury Prediction and Prevention (n=38)

Study	Year Country	Sport Population	Sample size	Study design	AI method	Injury type	Input variables	Performance metrics	Validation method	Key findings
Rossi et al. [18]	2018 Italy	Soccer Professional male	173	Retrospective cohort	RF	Non-contact injuries	GPS training load, locomotor patterns, and accumulated workload	85% precision	Temporal validation	RF identified high-risk training sessions based on movement patterns
Jauhiainen et al. [7]	2021 Finland	Basketball and floorball Youth athletes	314 (57 injuries)	Prospective cohort	L1-regularised logistic regression and Random Forest	Non-contact knee injuries	Wearable sensor time-series, biomechanical data from landing tests	Mean AUC-ROC 0.63 (range 0.51–0.69; best classifier: linear SVM)	Cross-validation	Identified knee abduction moment and centre-of-mass acceleration during cutting as candidate risk factors despite limited overall predictive power
Luu et al. [27]	2020 USA	Ice hockey (NHL) Professional male (2,109 position players; 213 goalies)	2,322	Retrospective cohort (10-year)	Five-algorithm comparison: RF, KNN, Naïve Bayes, XGBoost, Top three-ensemble; vs. logistic regression	Publicly reported next-season injuries	NA	XGBoost AUC 0.948 (position players); 0.956 (goalies)	Hold-out validation	XGBoost outperformed logistic regression for both subgroups; prior injury was the strongest predictor on SHAP analysis
Karnuta et al. [28]	2020 USA	Baseball (Major League Baseball) Professional male (1,931 position players;	13,982 player-years	Retrospective cohort (10-year)	Top three ensembles of Random Forest, KNN, Naïve Bayes, XGBoost, and logistic regression	Multiple injury types	Performance data, injury history, player characteristics	Mean AUC 0.76 (position players); 0.65 (pitchers)	Temporal validation	Ensemble ML modestly outperformed logistic regression for next-season MLB injury prediction in 13 of 14 comparisons

		1,245 pitchers)								
Min et al. [26]	2024 China	Basketball Collegiate athletes	158	Prospective	RF	Joint injuries	Biomechanical variables, training load metrics	85% accuracy; AUC 0.82	k-fold cross-validation	Demonstrated efficacy of ensemble methods; addressed class imbalance via SMOTE
Sobhani et al. [50]	2025 USA	Track & field Athletes	NR	NR	Support Vector Machine	Tibial stress injuries	NR	83% specificity	NR	SVM effectively distinguished injury risk categories
Calderón-Díaz et al. [29]	2023 Chile	Soccer Professional male	110	Prospective	Comparative evaluation of 35 ML configurations (XGBoost selected as best model)	Hamstring injuries	Biomechanical sensor data (sprint acceleration)	78% precision (XGBoost)	Holdout validation	Explainable ML identified at-risk athletes during high-intensity activities
Li et al. [51]	2023 China	Swimming Elite athletes	NR	Prospective	Statistical modelling/machine learning applied to HRV time-series data	Chronic insomnia and sleep continuity disruption as injury-related physiological markers	Heart rate variability, sleep quality data	Pre-sleep HRV identified as a significant predictor of chronic insomnia ($p < 0.05$) and impaired sleep continuity in national-level athletes	Cross-validation	Pre-sleep HRV predicted chronic insomnia and measures of sleep continuity in national-level athletes; physiological monitoring of recovery status identified as relevant to injury-related overtraining risk
Meng & Qiao [30]	2023 China	Multi-sport Collegiate athletes	NR	Prospective	Dual-feature neural network	Lower limb injuries	Biomechanical + physiological data	97% accuracy; 95.7% sensitivity; 97.5% specificity (internal evaluation)	NR	Reported high internal performance with dual-feature fusion neural network; no external validation or clinical-implementation arm
Ayala et al. [31]	2019 Spain	Soccer Professional male	NR	Retrospective	Decision tree (ADTree) base classifier with SMOTE oversampling and boosting ensembles	Hamstring injuries	Biceps femoris architectural factors	AUC 0.837; sensitivity 77.8%; specificity 83.8%	Temporal validation	Ensemble boosting on a decision-tree base outperformed comparator classifiers for hamstring strain injury risk identification
Lee Dow et	2021 Aus	Australian football	NR	Prospective	Logistic Regression	Hamstring injuries	Biceps femoris architectural risk	NR	External validation	Demonstrated transfer learning potential across similar sports

al. [32]	tralia	Professional male					factors (from soccer data)			
Rommers et al. [8]	2020 Belgium	Soccer Elite youth male	NR	Prospective	XGBoost, ML comparison	Non-contact injuries	GPS data, training load, biomechanics	AUC 0.82-0.89	Temporal validation	XGBoost superior for youth populations; addressed class imbalance
Piłka et al. [17]	2023 Poland	Soccer Professional male	173	Prospective	ML comparison (RF, SVM, others)	Multiple injury types	GPS-based wearable sensor data	Context-dependent (varied by algorithm)	Cross-validation	Highlighted context-specificity of algorithm performance
Kolodziej et al. [33]	2021 Germany	Soccer Elite youth male	62 (25 injuries)	Prospective	Classification & Regression Trees (CART)	Non-contact injuries	Neuromuscular performance parameters	NR	Temporal validation	Identified CMJ height <35 cm as risk threshold; enables direct intervention targeting
Windsor et al. [34]	2022 USA	American football Varsity athletes	NR	Retrospective	Feature selection methods	Foot biomechanical injuries	GPS training data, foot biomechanics	NR	NR	Applied systematic feature selection to identify predictive parameters
Liang et al. [35]	2023 China	Soccer Female players	43	Prospective	3D CNN with attention mechanisms	ACL injuries	3D motion capture data (jump-landing)	90% accuracy	Cross-validation	Explainable CNN highlighted specific movement components (knee valgus, landing mechanics)
VAN Hooren et al. [52]	2022 China	Distance running Collegiate runners	87	Prospective	Multimodal transformer	Tibial stress injuries, Achilles tendinopathy	Wearable sensor data + biomechanical assessments	AUC 0.91; 87% sensitivity; 85% specificity	Temporal validation	Attention-weighted feature fusion across data modalities; identified asymmetrical landing patterns during fatigue

Tabben et al. [54]	2026	Qatar	Football (soccer)	Professional male	n = 1,258 players; 4,700 injuries; 9 seasons (2013–2021)	Observational longitudinal prospective cohort	Markov chain probabilistic model	All time-loss injuries; subsequent/recurrent injuries (primary focus: hamstring and groin)	Injury body part, injury type (by category), injury recurrence sequence, within-season temporal structure	Subsequent injury rate 34% (1,599/4,700); hamstring within-season recurrence probability 7.5% ± 1.3%; groin-to-hamstring transition probability 2.9% ± 0.82%
Huang et al. [53]	2024 China	Badminton Elite athletes	NR	Prospective	Temporal graph convolutional network	Multiple injury types	Biomechanical, physiological, psychological factors	36% improvement vs. standard ML	NR	Modeled dynamic relationships; identified a 72- 96 h window between sleep disruption and biomechanical compensation
Li and Huang [36]	2024 China	Multi-sport Athletes	NR	Survey/narrative review	Comprehensive survey; NLP + ML hybrid (sentiment analysis of wellness reports as sub-finding)	Multiple injury types (survey-level synthesis)	Athletes' wellness reports (text data)	12% accuracy improvement vs. physiological data alone	Cross-validation	Comprehensive survey of AI and cloud computing applications in the sports industry; sentiment analysis of wellness text data reported to improve injury prediction accuracy by 12% over physiological data alone
Robertson et al. [37]	2022 England	Rugby union Professional male	696	Prospective multi-site	Federated learning	Multiple injury types	Playing surface, match data, training load	NR	Multi-site validation	Identification of playing surface as a significant modifier of match injury risk in professional rugby union over six seasons; multi-team surveillance data analysed using hierarchical statistical modelling to detect environment-related risk factors
Evans et al. [38]	2024 UK	Rugby union Professional male	NR	Prospective	Parallel ensemble averaging*	Non-contact lower limb injuries	Biomarkers (CK, cortisol), training load data	76% accuracy	Temporal validation	Identified physiological signatures preceding soft tissue injuries
Thornton et al. [39]	2019 Australia	Team sports (methodological) Not applicable	NR	Methodological/conceptual review	Statistical and visualisation frameworks (not an AI study)	Not applicable	Sleep quality, training load, GPS data	Not applicable	Temporal validation	Provided analytical and visualisation standards for athlete-monitoring systems
Johnson et al.	2025 USA	Multi-sport Collegiate athletes	219	Prospective	Multimodal deep learning	Multiple injury types	Environmental variables (surface, temperature), scheduling,	17% accuracy increase with environmental integration	Cross-validation	Environmental/situational variables enhance injury susceptibility modelling

[65]							contextual data			
Wang et al. [41]	2024 China	Basketball Male players	NR	Intervention study	CNN-based system	Landing-related injuries	Landing mechanics (video analysis)	37% reduction in high-risk landing mechanics	Pre-post comparison	Real-time biomechanical feedback informed personalised training
Arundale et al. [9]	2022 USA	Multi-sport Athletes post-ACL reconstruction	NR	Prospective intervention	Markerless motion capture + ML	ACL reinjury	Movement quality data (rehabilitation exercises)	28% reduction in reinjury rate (24-month follow-up)	Prospective cohort comparison	Immediate movement quality feedback during rehabilitation
Impellizzeri et al. [42, 43]	2020 Multiple	Soccer Professional athletes	NR	Theoretical framework	Reinforcement learning framework	Multiple injury types	Individual load-response models	NR	Simulation validation	Continuously updated individual load-response models for personalised recommendations
Van Eetvelde et al. [5]	2021 Multiple	Multi-sport Athletes	Systematic review	Systematic review	Digital twins (computational models)	Multiple injury types	Biomechanical, physiological, recovery data	NR	Review synthesis	Predicted individual responses to different training scenarios
Dallinga et al. [44]	2017 Netherlands	Multi-sport Male athletes	NR	RCT	Augmented reality + ML	Jump-landing injuries	Markerless motion capture, real-time feedback	Superior adherence and technique vs. conventional training	RCT	Real-time visual feedback enhanced jump-landing technique
Miri et al. [45]	2024	Basketball Athletes post-ACL	NR	Prospective intervention	Multi-task learning	ACL reinjury, compensation patterns	Rehabilitation monitoring data	42% reduction in reinjury rates	Prospective comparison	ML predicted reinjury probability and movement compensations

	Iran	reconstruction								
Clement et al. [67]	2024 Sweden	Multi-sport Athletes	NR	Prospective	AI-driven linguistic analysis	Psychological distress-related injury risk	Daily wellness reports (text data)	NR	Temporal validation	Monitored linguistic patterns to identify early psychological distress signs
Nassis et al. [15]	2023 Multiple	Soccer Professional athletes	NR	Implementation study	Multiple ML methods with CDSS	Multiple injury types	Multi-modal athlete monitoring data	Significantly higher adherence to prevention activities	Implementation comparison	Athlete-facing mobile app translated AI risk assessments into actionable recommendations
Ekstrand et al. [10]	2023 Europe	Soccer (men's professional)	21 seasons of data (2001/02–2021/22); up to 36 clubs per season	Prospective longitudinal cohort (UEFA Elite Club Injury Study)	Traditional epidemiological modelling; no AI method applied	Hamstring injuries (primary); all muscle injuries	Exposure hours (training and match), injury time-loss data, clinical diagnosis	Hamstring injuries constitute 24% of all injuries; injury rate increase documented across recent seasons	Longitudinal prospective cohort	Hamstring injury incidence increased significantly across 21 seasons; standardised UEFA surveillance methodology
Malone et al. [47]	2018 Multiple	Soccer Professional athletes	NR	Prospective	Integrated AI framework	Multiple injury types	High-speed running, sprinting, physical qualities	31% performance improvement; 26% injury reduction	Prospective comparison	Unified performance-injury framework superior to isolated approaches
West et al. [48]	2023 Multiple	Rugby union Youth athletes	Systematic review/meta-analysis	Systematic review	Multiple ML methods	Multiple injury types	Heterogeneous across studies	Injury definition variations accounted for 37% of model performance differences	Review synthesis	Highlighted critical need for standardised injury definitions
Li et al. [49]	2024 Chi	Multi-sport Athletes	NR	Qualitative/theoretical	Data rights framework	N/A (data governance focus)	Athlete health data governance	N/A	Conceptual analysis	Examined privacy concerns, consent, and data usage in athlete monitoring

	na									
Claudino et al. [11]	2019 Multiple	Team sports Athletes	Systematic review	Systematic review	Multiple ML methods	Multiple injury types	Heterogeneous across studies	AUC 0.70 (early applications)	Review synthesis	Early systematic review identifying methodological limitations
Cust et al. [22]	2019 Australia	Multi-sport Athletes	Systematic review	Systematic review	ML and DL methods	Movement recognition for injury risk	Heterogeneous across studies	AUC 0.79 (ensemble methods)	Review synthesis	Examined ML/DL for sport-specific movement recognition
Desai [20]	2024 USA	Multi-sport Athletes	Narrative review	Narrative review	Multiple AI methods	Multiple injury types (focus on return-to-play)	Multimodal data	Stacked ensembles outperformed single multimodal models	Review synthesis	Future directions in AI for sports medicine and return-to-play

ACL = Anterior Cruciate Ligament; ANN = Artificial Neural Network; AUC = Area Under the Curve; CART = Classification and Regression Trees; CDSS = Clinical Decision Support System; CK = Creatine Kinase; CMJ = Countermovement Jump; CNN = Convolutional Neural Network; DL = Deep Learning; GPS = Global Positioning System; HRV = Heart Rate Variability; KNN = K-Nearest Neighbors; LSTM = Long Short-Term Memory; ML = Machine Learning; N/A= Not Applicable; NLP = Natural Language Processing; NR = Not Reported; RCT = Randomized Controlled Trial; RF = Random Forest; SMOTE = Synthetic Minority Oversampling Technique; SVM = Support Vector Machine; XGBoost = Extreme Gradient Boosting; 3D = Three-Dimensional.

Supplementary Table S3. Structured methodological quality assessment of included studies based on adapted PROBAST and TRIPOD frameworks (n = 38).

Manuscript: Application of Artificial Intelligence for Predicting Sports Injuries and Customizing Personalised Prevention Strategies: A Scoping Review (bioengineering-4346431)

Study (Author [Ref])	Study design	Sample size (n)	AI/ML/DL method	Validation strategy	External validation	Performance metrics reported	Domain 1: Clarity of objectives	Domain 2: Methodology appropriateness	Domain 3: Limitations discussed	Overall quality
Rossi et al. [18]	Retrospective cohort	173	Random Forest	Temporal (hold-out season)	No	Partial (precision only; 85%)	Adequate	Adequate	Adequate	Partial
Jauhiainen et al. [7]	Prospective cohort	314	L1-LR; Random Forest; Linear SVM	k-fold cross-validation	No	Adequate (AUC-ROC 0.63, range)	Adequate	Adequate	Adequate	Adequate
Luu et al. [27]	Retrospective cohort (10-yr)	2,322	XGBoost; RF; KNN; Naïve Bayes; LR (5-algorithm ensemble)	Hold-out validation	No	Adequate (AUC 0.948–0.956)	Adequate	Adequate	Adequate	Adequate
Karnuta et al. [28]	Retrospective cohort (10-yr)	13,982 player-yrs	Ensemble: RF; KNN; Naïve Bayes; XGBoost; LR	Temporal validation	No	Adequate (AUC 0.65–0.76)	Adequate	Adequate	Adequate	Adequate
Min et al. [26]	Prospective cohort	158	Random Forest	k-fold cross-validation	No	Adequate (accuracy 85%; AUC 0.82)	Adequate	Adequate	Adequate	Adequate
Sobhani et al. [50]	Cross-sectional (NR)	NR	Support Vector Machine	NR	NR	Partial (specificity 83% only)	Partial	Adequate	Partial	Partial
Calderón-Díaz et al. [29]	Prospective cohort	110	XGBoost (best of 35 ML configs)	Hold-out validation	No	Partial (precision 78% only)	Adequate	Adequate	Adequate	Adequate

Li et al. [51]	Prospective cohort	NR	Statistical modelling (HRV analysis; machine learning applied to physiological time-series)	k-fold cross-validation	No	Partial (p-values and regression coefficients reported; no AUC or sensitivity/specificity)	Adequate	Adequate	Partial	Partial
Meng & Qiao [30]	Prospective cohort	NR	Dual-feature fusion neural network	None (internal evaluation)	No	Adequate (acc 97%; sens 95.7%; spec 97.5%)	Adequate	Adequate	Partial	Partial
Ayala et al. [31]	Retrospective cohort	NR	ADTree + SMOTE + boosting ensemble	Temporal validation	No	Adequate (AUC 0.837; sens 77.8%; spec 83.8%)	Adequate	Adequate	Adequate	Adequate
Lee Dow et al. [32]	Prospective cohort	NR	Logistic Regression	External (independent cohort)	Yes	Partial (no AUC reported)	Adequate	Adequate	Adequate	Adequate
Rommers et al. [8]	Prospective cohort	NR	XGBoost (ML algorithm comparison)	Temporal validation	No	Adequate (AUC 0.82–0.89)	Adequate	Adequate	Adequate	Adequate
Tabben et al. [54]	Observational longitudinal cohort	1,258 (4,700 injuries; 9 seasons)	Markov chain probabilistic model	Internal validation (95% CI on transition probability estimates; nine-season longitudinal dataset)	No	Adequate (transition probabilities with 95% CI; recurrence rates with standard error reported)	Adequate	Adequate	Adequate	Adequate
Piłka et al. [17]	Prospective cohort	173	RF; SVM; others (comparison)	k-fold cross-validation	No	Partial (context-dependent; varied)	Adequate	Adequate	Adequate	Adequate
Kolodziej et al. [33]	Prospective cohort	62 (25 injuries)	CART	Temporal validation	No	Partial (threshold only; no AUC)	Adequate	Adequate	Partial	Partial
Windsor et al. [34]	Retrospective cohort	NR	Feature selection (GPS variables)	NR	NR	Inadequate (NR)	Partial	Partial	Partial	Inadequate
Liang et al. [35]	Prospective cohort	43	3D CNN + dual attention mechanisms	5-fold cross-validation	No	Adequate (acc 80.6%; AUC 0.889)	Adequate	Adequate	Adequate	Adequate
VAN Hooren et al. [52]	Prospective cohort	87	Multimodal transformer	Temporal validation	No	Adequate (AUC 0.91; sens 87%; spec 85%)	Adequate	Adequate	Adequate	Adequate

Huang et al. [53]	Prospective cohort	NR	Temporal graph convolutional network	NR	No	Partial (36% improvement vs ML)	Adequate	Adequate	Partial	Partial
Li and Huang [36]	Prospective cohort	NR	NLP + ML hybrid (sentiment analysis)	k-fold cross-validation	No	Partial (12% accuracy improvement)	Adequate	Adequate	Partial	Partial
Robertson et al. [37]	Prospective multi-site	696	Federated learning	Multi-site validation	Yes	Partial (metrics NR; qualitative)	Adequate	Adequate	Adequate	Adequate
Evans et al. [38]	Prospective cohort	NR	Parallel ensemble averaging	Temporal validation	No	Partial (accuracy 76% only)	Adequate	Adequate	Adequate	Partial
Thornton et al. [39]	Methodological review	NA	Statistical/visualisation frameworks (not AI)	Temporal validation	NA	NA (methodological; no prediction)	Adequate	Partial	Adequate	Partial
Johnson et al. [65]	Prospective cohort	219	Multimodal deep learning	k-fold cross-validation	No	Partial (17% accuracy improvement)	Adequate	Adequate	Adequate	Adequate
Wang et al. [41]	Intervention study	NR	CNN-based system (video analysis)	Pre-post comparison	No	Partial (37% reduction in risk mechanics)	Adequate	Adequate	Adequate	Partial
Arundale et al. [9]	Prospective intervention	NR	Markerless motion capture + ML	Prospective cohort comparison	No	Partial (28% reinjury reduction)	Adequate	Adequate	Adequate	Partial
Impellizzeri et al. [42,43]	Theoretical framework	NA	Reinforcement learning framework	Simulation validation	NA	Inadequate (NR)	Adequate	Partial	Adequate	Partial
Van Eetvelde et al. [5]	Systematic review	Multiple	Digital twins (computational models)	Review synthesis	NA	Inadequate (NR)	Adequate	Partial	Adequate	Partial
Dallinga et al. [44]	Randomised controlled trial	NR	Augmented reality + ML	RCT	No	Partial (adherence + technique)	Adequate	Adequate	Adequate	Partial
Miri et al. [45]	Prospective intervention	NR	Multi-task learning	Prospective cohort comparison	No	Partial (42% reinjury reduction)	Adequate	Adequate	Adequate	Partial

Clement et al. [67]	Prospective cohort	NR	AI-driven linguistic analysis (NLP)	Temporal validation	No	Inadequate (NR)	Adequate	Partial	Partial	Partial
Nassis et al. [15]	Implementation study	NR	Multiple ML methods + CDSS	Implementation comparison	No	Partial (adherence outcomes only)	Adequate	Adequate	Adequate	Partial
Ekstrand et al. [10]	Prospective longitudinal cohort (UEFA ECIS)	21 seasons (multiple clubs)	Traditional epidemiological analysis	Longitudinal cohort surveillance	No	Partial (injury incidence rates reported; no prediction model performance metrics)	Adequate	Partial	Adequate	Partial
Malone et al. [47]	Prospective cohort	NR	Integrated AI framework	Prospective comparison	No	Partial (% outcome metrics)	Adequate	Adequate	Adequate	Partial
West et al. [48]	Systematic review/meta-analysis	Multiple	Multiple ML methods	Review synthesis	NA	Partial (effect size reported)	Adequate	Partial	Adequate	Partial
Li et al. [49]	Qualitative/theoretical	NR	Data rights framework	Conceptual analysis	NA	NA (data governance focus)	Adequate	Partial	Adequate	Partial
Claudino et al. [11]	Systematic review	Multiple	Multiple ML methods	Review synthesis	NA	Partial (AUC 0.70)	Adequate	Partial	Adequate	Partial
Cust et al. [22]	Systematic review	Multiple	ML and DL methods	Review synthesis	NA	Partial (AUC 0.79)	Adequate	Partial	Adequate	Partial
Desai [20]	Narrative review	Multiple	Multiple AI methods	Review synthesis	NA	Partial (comparative)	Adequate	Partial	Adequate	Partial

Aggregate results (n = 39). Domain 1 — Clarity of objectives: Adequate 35/39 (90%), Partial 4/39 (10%), Inadequate 0/39 (0%). Domain 2 — AI methodology appropriateness: Adequate 30/39 (77%), Partial 9/39 (23%), Inadequate 0/39 (0%). Domain 3 — Limitations discussed: Adequate 32/39 (82%), Partial 7/39 (18%), Inadequate 0/39 (0%). External validation: Yes 5/39 (13%), No 24/39 (62%), NA/NR 10/39 (26%). Overall quality: Adequate 15/39 (38%), Partial 23/39 (59%), Inadequate 1/39 (3%).

Abbreviations. 3D = Three-Dimensional; **ACL** = Anterior Cruciate Ligament; **AI** = Artificial Intelligence; **AUC** = Area Under the Curve; **CART** = Classification and Regression Trees; **CDSS** = Clinical Decision Support System; **CNN** = Convolutional Neural Network; **DL** = Deep Learning; **GPS** = Global Positioning System; **KNN** = K-Nearest Neighbors; **LR** = Logistic Regression; **LSTM** = Long Short-Term Memory; **ML** = Machine

Learning; **NA** = Not Applicable; **NLP** = Natural Language Processing; **NR** = Not Reported; **PROBAST** = Prediction Model Risk of Bias Assessment Tool; **RCT** = Randomised Controlled Trial; **RF** = Random Forest; **SMOTE** = Synthetic Minority Oversampling Technique; **sens** = sensitivity; **spec** = specificity; **SVM** = Support Vector Machine; **TRIPOD** = Transparent Reporting of a multivariable prediction model for Individual Prognosis or Diagnosis; **XGBoost** = Extreme Gradient Boosting.

Domain rating criteria. Ratings were applied to three domains of the adapted PROBAST/TRIPOD framework (see §2.6). Domain 1 — Clarity of objectives: Adequate = research question, prediction target, and target population explicitly stated; Partial = objective inferable but not formally stated; Inadequate = absent or ambiguous. Domain 2 — AI/ML/DL methodology appropriateness: Adequate = algorithm choice aligned with data type and prediction task (e.g., tree-based for tabular data; CNN/RNN for time-series or image inputs) with justification provided; Partial = minor mismatch or insufficient justification; Inadequate = clear mismatch between method and data structure. Domain 3 — Limitations discussed: Adequate = dedicated limitations section with specific methodological acknowledgements (e.g., small sample size, class imbalance, absence of external validation); Partial = brief mention without elaboration; Inadequate = absent.

Performance metrics (reported column). Adequate = AUC reported alongside at least one of sensitivity, specificity, or accuracy; Partial = single metric only, or comparative percentage improvement only; Inadequate = not reported; NA = not applicable (review articles, governance studies, and methodological frameworks for which individual model performance reporting is not the primary output).

External validation column. Yes = independent cohort validation formally conducted and reported; No = not conducted; NA = not applicable (review articles, theoretical frameworks, methodological studies); NR = not reported.

Overall quality rating. Adequate = three of three domains rated Adequate; Partial = one or two domains rated Adequate; Inadequate = no domain rated Adequate. Note: studies rated Partial or Inadequate are not excluded from the synthesis, and the rating does not reflect scientific merit per se; it reflects the completeness of methodological reporting and validation relative to current prediction-model standards (PROBAST: Moons et al., 2019; TRIPOD: Collins et al., 2015).

References for quality framework.

Moons, K. G. M., et al. (2019). PROBAST: A tool to assess risk of bias and applicability of prediction model studies: Explanation and elaboration. Annals of Internal Medicine, 170(1), W1–W33. https://doi.org/10.7326/M18-1377

Collins, G. S., et al. (2015). Transparent reporting of a multivariable prediction model for individual prognosis or diagnosis (TRIPOD): The TRIPOD statement. BMC Medicine, 13, Article 1. https://doi.org/10.1186/s12916-014-0241-z

Supplementary Material. Full Boolean Search Strings and Database-Specific Syntax

Manuscript: Application of Artificial Intelligence for Predicting Sports Injuries and Customizing Personalised Prevention Strategies: A Scoping Review (bioengineering-4346431)

Search date: Through February 2026. No lower date restriction applied.

Database 1: PubMed (with MeSH terms)

("artificial intelligence"[MeSH] OR "machine learning"[MeSH] OR "deep learning"[tiab] OR
"neural networks, computer"[MeSH] OR "neural network"[tiab] OR "random forest"[tiab] OR
"support vector machine"[tiab] OR "gradient boosting"[tiab] OR "XGBoost"[tiab] OR
"convolutional neural network"[tiab] OR "recurrent neural network"[tiab] OR
"long short-term memory"[tiab] OR "LSTM"[tiab] OR
"explainable artificial intelligence"[tiab] OR "federated learning"[tiab] OR
"expert systems"[MeSH])
AND
("athletic injuries"[MeSH] OR "sports injuries"[tiab] OR "athletic injury"[tiab] OR
"musculoskeletal injury"[tiab] OR "acute injury"[tiab] OR "overuse injury"[tiab])
AND
("risk assessment"[MeSH] OR "prediction"[tiab] OR "prevention and control"[MeSH Subheading] OR
"injury prevention"[tiab] OR "risk assessment"[tiab] OR "risk stratification"[tiab])

Database 2: Web of Science (Core Collection)

TS=("artificial intelligence" OR "machine learning" OR "deep learning" OR
"neural network*" OR "random forest" OR "support vector machine" OR
"gradient boosting" OR "XGBoost" OR "convolutional neural network" OR
"recurrent neural network" OR "LSTM" OR "long short-term memory" OR
"explainable AI" OR "federated learning" OR "expert system*")
AND
TS=("sport* injur*" OR "athletic injur*" OR "musculoskeletal injur*" OR
"acute injur*" OR "overuse injur*")
AND
TS=("prediction" OR "prevention" OR "risk assessment" OR "injury prevention" OR
"risk stratification")

Database 3: IEEE Xplore

("artificial intelligence" OR "machine learning" OR "deep learning" OR
"neural network" OR "random forest" OR "support vector machine" OR
"convolutional neural network" OR "recurrent neural network" OR "LSTM" OR
"gradient boosting" OR "XGBoost" OR "federated learning" OR "explainable AI")

AND
("sports injury" OR "athletic injury" OR "musculoskeletal injury" OR
"biomechanical risk" OR "injury prediction")
AND
("prediction" OR "prevention" OR "risk assessment" OR "wearable sensor" OR
"motion capture")

Database 4: Scopus

```
TITLE-ABS-KEY(
  ("artificial intelligence" OR "machine learning" OR "deep learning" OR
  "neural network" OR "random forest" OR "support vector machine" OR
  "convolutional neural network" OR "recurrent neural network" OR "LSTM" OR
  "XGBoost" OR "gradient boosting" OR "federated learning" OR
  "explainable artificial intelligence")
  AND
  ("sports injur*" OR "athletic injur*" OR "musculoskeletal injur*" OR
  "overuse injur*" OR "acute injur*")
  AND
  ("prediction" OR "prevention" OR "risk assessment" OR "injury prevention")
)
```

Database 5: Google Scholar — Sampling Protocol

Per the method of Haddaway et al. (2015), Google Scholar was searched using the query strings below. Screening was bounded to the first 200 records returned, ranked by relevance, for each query string. All retrieved records were subjected to the same title-and-abstract eligibility assessment applied to records from structured databases. To avoid personalisation of results, all searches were conducted in a logged-out browser session.

Query 1:

"machine learning" "sports injury" prediction prevention

Query 2:

"deep learning" "athletic injury" risk assessment

Query 3:

"artificial intelligence" "injury prediction" sport athletes

Query 4:

"neural network" "sports injury" prevention biomechanical

Query 5:

"random forest" OR "XGBoost" "injury risk" sport

Records identified via Google Scholar that duplicated records already retrieved from PubMed, Web of Science, IEEE Xplore, or Scopus were removed at the deduplication stage. Google Scholar contributed n = 122 unique records after deduplication (see Figure 1, PRISMA 2020 flow diagram).

Abbreviations used in this supplementary material: AI = Artificial Intelligence; DL = Deep Learning; LSTM = Long Short-Term Memory; MeSH = Medical Subject Headings (PubMed); ML = Machine Learning; PRISMA = Preferred Reporting Items for Systematic Reviews and Meta-Analyses; tiab = title and abstract field tag (PubMed); TS = topic search field tag (Web of Science); XGBoost = Extreme Gradient Boosting.

Reference: Haddaway, N. R., Collins, A. M., Coughlin, D., & Kirk, S. (2015). The role of Google Scholar in evidence reviews and its applicability to grey literature searching. *PLOS ONE, 10*(9), Article e0138237. https://doi.org/10.1371/journal.pone.0138237
